# Endocytosis as a Biological Response in Receptor Pharmacology: Evaluation by Fluorescence Microscopy

**DOI:** 10.1371/journal.pone.0122604

**Published:** 2015-04-07

**Authors:** Víctor M. Campa, Almudena Capilla, María J. Varela, Arlet M. Acanda de la Rocha, Juan C. Fernandez-Troyano, R. Belén Barreiro, Juan F. Lopez-Gimenez

**Affiliations:** 1 Institute of Biomedicine and Biotechnology of Cantabria, (IBBTEC), CSIC, UC, Santander, Spain; 2 Department of Biological and Health Psychology, Autonoma University of Madrid (UAM), Madrid, Spain; 3 Center for Research in Molecular Medicine and Chronic Diseases (CIMUS), University of Santiago de Compostela (USC), Santiago de Compostela, Spain; 4 Institute of Physics of Cantabria, (IFCA), CSIC, UC, Santander, Spain; University of Cologne, GERMANY

## Abstract

The activation of G-protein coupled receptors by agonist compounds results in diverse biological responses in cells, such as the endocytosis process consisting in the translocation of receptors from the plasma membrane to the cytoplasm within internalizing vesicles or endosomes. In order to functionally evaluate endocytosis events resulted from pharmacological responses, we have developed an image analysis method –the Q-Endosomes algorithm– that specifically discriminates the fluorescent signal originated at endosomes from that one observed at the plasma membrane in images obtained from living cells by fluorescence microscopy. Mu opioid (MOP) receptor tagged at the carboxy-terminus with yellow fluorescent protein (YFP) and permanently expressed in HEK293 cells was used as experimental model to validate this methodology. Time-course experiments performed with several agonists resulted in different sigmoid curves depending on the drug used to initiate MOP receptor endocytosis. Thus, endocytosis resulting from the simultaneous activation of co-expressed MOP and serotonin 5-HT_2C_ receptors by morphine plus serotonin was significantly different, in kinetics as well as in maximal response parameters, from the one caused by DAMGO, sufentanyl or methadone. Therefore, this analytical tool permits the pharmacological characterization of receptor endocytosis in living cells with functional and temporal resolution.

## Introduction

Endocytosis of G-protein coupled receptors (GPCRs) is a cellular event that generally occurs upon receptor activation by agonist compounds. Essentially, the process consists in the formation and inward cytosolic movement of vesicles originated at the plasma membrane. As a consequence, activated receptors abandon the cell surface within these vesicles, termed endosomes, to follow different sorting pathways leading them to degradation or recycling back to the plasma membrane [1]. In fact, endocytosis has been traditionally considered as a mechanism to interrupt the different intracellular signaling cascades initiated by receptor activation, although that widely accepted paradigm has been brought into question after a recent report demonstrating β2 adrenoceptor intracellular signaling activity from endosomes [2].

Fluorescence microscopy technology has made feasible to observe receptor endocytosis at subcellular resolution. Moreover, the development of recombinant DNA technologies has permitted to tag receptors with fluorescent proteins in order to make them visible by fluorescence microscopy after their heterologous expression in cell lines. In this model, both receptor and fluorescent protein constitute a single fusion protein possible to be observed throughout the different cellular compartments without the participation of additional labelling elements such as antibodies or fluorescent ligands [3]. One of the main advantages of this methodology is the possibility of carrying out imaging experiments with living cells and to observe, for instance, with high spatial and temporal resolution the formation of endocytic vesicles and their subsequent internalisation from the plasma membrane to the cytoplasm in real time. By using this experimental approach, we previously described that coactivation of serotonin 5-HT_2A_ receptor facilitates morphine internalization of the mu opioid receptor (MOP) expressed in a heterologous/inducible cell system [4].

The faculty of an agonist to promote a response through a given receptor in a specific tissue is known as efficacy. This attribute is inherent to the chemical nature of the compound, relative when comparing agonist drugs to each others and, interestingly, it depends on the type of response considered for evaluation, i.e., some agonists presenting a significant efficacy for a particular response may be incompetent to evoke a reaction to a different functional effect. This later property, which is known in pharmacology as biased agonism or functional selectivity, is defined as the ability of different agonists for a particular receptor to differentially activate either signalling cascades or regulatory events, including differences in receptor trafficking [5]. Mu opioid (MOP) receptor constitutes a patent example of functional selectivity in relation to the capacity of some agonists to promote endocytosis upon receptor activation [6]; thus, while agonists such as DAMGO (Tyr-D-Ala-Gly-N-Met-Phe-Gly-ol) and morphine display intrinsic activity in both G-protein coupling ([^35^S]GTPγS binding) and adenylyl cyclase inhibition assays, they are completely disparate in terms of generating receptor internalization since morphine is incapable to induce MOP receptor endocytosis in the majority of cellular systems [7–9]. There are numerous experimental data supporting the hypothesis that associates the inefficiency of morphine to induce endocytosis of MOP receptors at the cellular level with the high degree of tolerance to antinociceptive effects developed after morphine sustained treatment (for a review on this topic see [8–10]). According to this, investigations on experimental conditions facilitating the internalization of MOP receptors by morphine would be an objective in the direction to model new therapeutic strategies for treatment of chronic pain.

Receptor pharmacology is a quantitative discipline, and therefore any biological effects generated as a result of ligand-receptor interactions should be monitored by analytical means providing measurable magnitudes corresponding to pharmacological properties of those compounds, i.e., affinity to bind to a specific site and/or potency and efficacy to evoke a biological response. In this respect, however, there is a deficiency of quantitative methods to accurately evaluate the functionality of GPCRs in terms of agonist efficacy to promote endocytosis. For this reason, we have created an algorithm that detects and measures endosomes by analysing images obtained from living cells by epifluorescence microscopy in real time. We opted for MOP receptor fused to YFP at the carboxyl terminus and permanently expressed in HEK 293 cells as a experimental model for validating our methodology because the following reasons: firstly, in basal conditions MOP receptors are predominantly located at the plasma membrane in these host cells and secondly, the existence of agonist drugs for this receptor clearly differentiated among them in terms of their capacity to induce MOP receptor internalization. In addition, the versatility of this experimental approach has been tested with another GPCR, i.e. delta opioid receptor fused to YFP (DOP-YFP) as well as by using host cells presenting different phenotypic characteristics.

## Materials and Methods

### Materials

All materials for tissue culture were from Life Technologies. Doxycycline, serotonin (5-HT), DTLET and DAMGO, were from Sigma-Aldrich. (±)DOI and WAY161503 were supplied by Tocrys Bioscience. Morphine and methadone was obtained from Alcaliber and sufentanyl was generously gifted by Prof. María del Amor Hurlé from the Department of Pharmacology and Physiology (University of Cantabria).

### Receptor Fusions with Fluorescent Proteins

Generation and subcloning of the human 5-HT_2C_ receptor construct was essentially as described previously (4). Briefly, the c-myc epitope tag was added at the amino terminus of the receptor by PCR techniques using forward primers containing the sequence of the c-myc epitope (amino acid sequence EQKLISEEDL). A c-myc-5-HT_2C_ receptor C-terminally tagged with a version of the enhanced cyan fluorescent protein (eCFP) called Cerulean was constructed by amplifying the sequence corresponding to the receptor by PCR and by removing the stop codon. This PCR product was ligated to the fluorescent protein sequence amplified by PCR and containing the same endonuclease restriction site (NotI). The final product of this ligation corresponds to a single open reading frame encoding the receptor-fluorescent protein fusion. c-myc-5-HT_2C_-Cerulean was subcloned into the vector pcDNA5/FRT/TO (Life Technologies) for the subsequent generation of Flp-In T-REx HEK293 cell lines. MOP-YFP receptor was obtained after amplification of human MOP using PCR primers containing a SacI endonuclease site at the 5′ end and an ApaI endonuclease site at the 3′ end and in the process removing the stop codon. This PCR product was subcloned into peYFP-N1 vector, resulting in a single open reading frame consisting of the MOP receptor with eYFP (enhanced Yellow Fluorescent Protein) fused to the receptor carboxyl terminus. DOP-YFP receptor construct was engineered following a similar experimental strategy. All the constructs were verified by DNA sequencing.

### Generation of Stable Flp-In T-REx HEK293 Cell Lines

To generate Flp-In T-REx HEK293 cell lines able to express c-myc-5-HT_2C_-Cerulean in an inducible manner, cells were transfected with a mixture containing the c-myc-5-HT_2A_-Cerulean receptor cDNA in the pcDNA5/FRT/TO vector and pOG44 vector (1:9) using the polyethylenimine method described elsewhere [24]. When cotransfected with the pcDNA5/FRT plasmid into the Flp-In mammalian host cell line, the Flp recombinase expressed from pOG44 mediates integration of the pcDNA5/FRT vector containing the gene of interest into the genome via Flp recombination target (FRT) sites. Cell maintenance and selection were as described [25]. Clones resistant to hygromycin were screened for c-myc-5-HT_2C_-Cerulean expression by both fluorescence and Western blotting. To induce expression of c-myc-5-HT_2C_-Cerulean cells were treated with varying concentrations of doxycycline for different periods. The optimal expression of c-myc-5-HT_2C_-Cerulean was achieved after 24-h treatment with 0.01 μg of doxycycline/ml growth medium. A double stable cell line expressing MOP-eYFP constitutively and c-myc-5-HT_2C_-Cerulean in an inducible manner was generated from the Flp-In T-REx HEK293 cells described above. Cells were transfected using the polyethylenimine method with the vector containing MOP-eYFP. Following transfection, cells were selected for resistance to Geneticin (G418; 1 mg/ml; InvivoGen), and the resistant clones were screened for receptor expression by fluorescence microscopy. Dialyzed fetal calf serum was used for cell growth to avoid activation of c-myc-5-HT_2C_-Cerulean by 5-HT that is present routinely in serum.

### Cell Transient Transfection

Flp-In T-REx HEK293 and HeLa cells were maintained in Dulbecco’s modified Eagle’s medium supplemented with 0.292 g/l L-glutamine and 10% (v/v) newborn calf serum at 37°C in a 5% CO2 humidified atmosphere. Cells were grown to 60–80% confluence before transient transfection. Transfection was performed by the polyethylenimine method (24).

### Living Cell Confocal Microscopy

Cells expressing receptors tagged with Cerulean or eYFP were grown on poly-d-lysine-treated coverslips. The coverslips were placed into a microscope chamber containing physiological saline solution (130 mM NaCl, 5 mM KCl, 1 mM CaCl_2_, 1 mM MgCl_2_, 20 mM HEPES, and 10 mM d-glucose, pH 7.4). Confocal images (512x512 pixels; 0.15m pixel size) were acquired at room temperature in a SP-5 laser-scan confocal microscope (Leica Microsystems) with a 40x 1.25NA oil objective, a 1 Airy pinhole, a 5x electronic zoom and 200Hz speed. Cells were excited sequentially with 548nm and 514nm laser lines and emission captured between 465-511nm (CFP) and 525-575nm (YFP). Images are presented after digital adjustment of brightness and contrast to maximize signal.

### Living Cell Epifluorescence Microscopy

Flp-In T-REx HEK293 permanently expressing MOP-YFP receptors and treated for 24 hours with doxycycline [0.01 μg/ml] to induce the expression of c-myc-5-HT_2C_-Cerulean receptors were grown on poly-d-lysine-treated coverslips. The coverslips were placed into a microscope chamber containing physiological saline solution (130 mM NaCl, 5 mM KCl, 1 mM CaCl_2_, 1 mM MgCl_2_, 20 mM HEPES, and 10 mM d-glucose, pH 7.4). For internalization experiments in real time, drugs diluted in physiological saline solution were perfused into the microscope chamber and ≈7 m thick Z-stacks (15 planes of 0.49 m Z-step size) were acquired for 15 minutes at a rate of 1 frame per minute in an inverted epifluorescence microscope (AF6500; Leica Microsystems) equipped with temperature control (PeCon GmbH) at 37ºC. Cell fluorescence were visualized with a 63x 1.3NA oil objective, 470/40 excitation and 515LP emission filters, and images (1004x1002 pixels; 0.13m pixel size) were acquired with a DU8285_VP (Andor Technology) camera using exposures of 200ms or less and an EM Gain of 20.

### Algorithm for Detecting and Quantifying Endosomes

In order to quantify endosomes we have designed the Q-Endosomes algorithm in Matlab that selectively identifies high intensity local maxima with the typical shape of an endosome (S1 Q_Endosomes). Fluorescence signal from endosomes is characterized by a 2D-Gaussian shape of a given width indicated by the parameter sigma (σ). A 2D-Gaussian function of unity amplitude, with the same sigma value in both *x* and *y* axis, and centered at 0, can be expressed as:

$$g\left(x,y\right)\;=\;e^{-\frac{x^{2}+y^{2}}{2\sigma^{2}}}$$

The algorithm consists of the following steps that are iteratively repeated for the data acquired at each time point. Firstly, each z-plane image (1004x1002 pixels) is filtered with a 2D-Gaussian of sigma 1 pixel (0.13 μm). This step aims to reduce instrumental noise resulting in smoother images. Secondly, to obtain a 2D projection of the 3D data, the maximum value across z-planes is selected for each pixel, i.e. z-projection. Thirdly, the algorithm identifies all local maxima that are a given percent above the local background (by default 90%). Hence, only local maxima with a relatively high intensity are selected as candidates. Finally, a 9x9 pixel region centered at each candidate is fitted to a 2D-Gaussian function of sigma 2.30 pixels (0.29 μm), corresponding to the width of an average real endosome. The value of this later parameter was experimentally determined, and it is critical to distinguish the fluorescence emitted by endosomes from the plasma membrane fluorescence since the distinctive sigma of the membrane is significantly higher (i.e. 5.15 pixels, see S1C Fig). A candidate local maxima is thus considered a real endosome if the correlation with the abovementioned 2D-Gaussian function is higher than 0.75 (see S2 Fig, S1 Supplemental Data for algorithm details and Matlab code in S1 Q_Endosomes).

The accuracy of the algorithm was assessed by using a model where a known number of simulated endosomes were randomly placed. These endosomes had the same features, in terms of sigma and intensity distribution, than a sample of manually identified endosomes (N = 150, sigma = 2.07 ± 0.53 pixels before smoothing, intensity = 1652 ± 178 arbitrary fluorescence units; mean ± SD). Accuracy, defined as the proportion of endosomes correctly detected, was higher than 90% for a model comprising between 0 and 800 simulated endosomes. (See S1 Supplemental Data).

### Quantitative Analysis of Endocytosis by Sigmoid Function Fitting

The kinetics of the number of endosomes generated upon agonist stimulation was analyzed using Prism (GraphPad Software Inc.). Quantification of endocytosis in terms of number of endosomes as a function of time was fitted to a sigmoid function according to the following equation:

$$y\;=\;bottom+\frac{\left(top-bottom\right)}{1+10^{\left[\left(time1/2-X\right)*slope\right]}}$$

When comparing parameters from different experimental conditions statistical significance was determined using an ANOVA followed by the post hoc analysis as appropriate. *P* < 0.05 determined statistical significance.

## Results and Discussion

**Fig 1:**
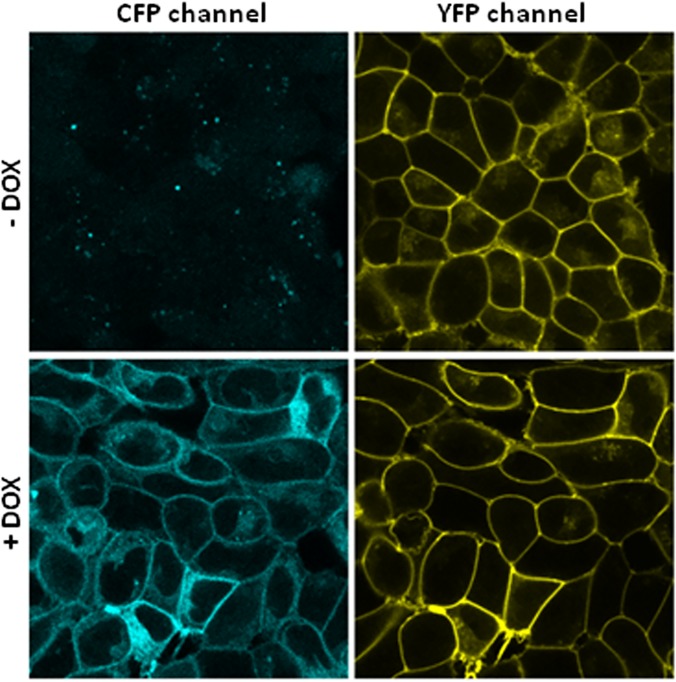
Generation of an inducible double-stable cell line expressing MOP-YFP and c-myc-5-HT_2C_-Cerulean receptors. Images obtained by confocal microscopy from living Flp-In HEK293 cells expressing permanently MOP-YFP receptors and c-myc-5-HT_2C_-Cerulean receptors in an inducible manner. The right column corresponds to the images resulting after exciting the cells with the YFP light settings whereas in the left column are the same microscopy field illuminated for CFP visualization. When indicated, +DOX corresponds to treatment with doxycycline (0.01 μg/ml) for 24 hours prior to microscope observation.

Similarly as described earlier [4], we generated a double stable Flp-In HEK293 cell line permanently expressing MOP-YFP receptors and harbouring serotonin c-myc-5-HT_2C_-Cerulean receptors in the inducible locus. Images obtained from these cells by confocal microscopy show a homogenous distribution of MOP-YFP receptors essentially confined to the plasma membrane when samples were illuminated to excite YFP light emission (Fig 1). Equivalent microscopic fields illuminated to visualize CFP (Cerulean) resulted in a complete absence of specific fluorescent signal (Fig 1). On the other hand, specific CFP signal corresponding to Cerulean protein was detected when cells were pre-treated with doxycycline for 24 hours to induce the expression of c-myc-5-HT_2C_-Cerulean receptors (Fig 1). The cellular distribution of MOP receptors was not affected by doxycycline treatment, and although c-myc-5-HT_2C_-Cerulean receptors are also expressed in intracellular compartments, both opioid and serotonin receptors seem to colocalize at the plasma membrane level (Fig 1).

**Fig 2:**
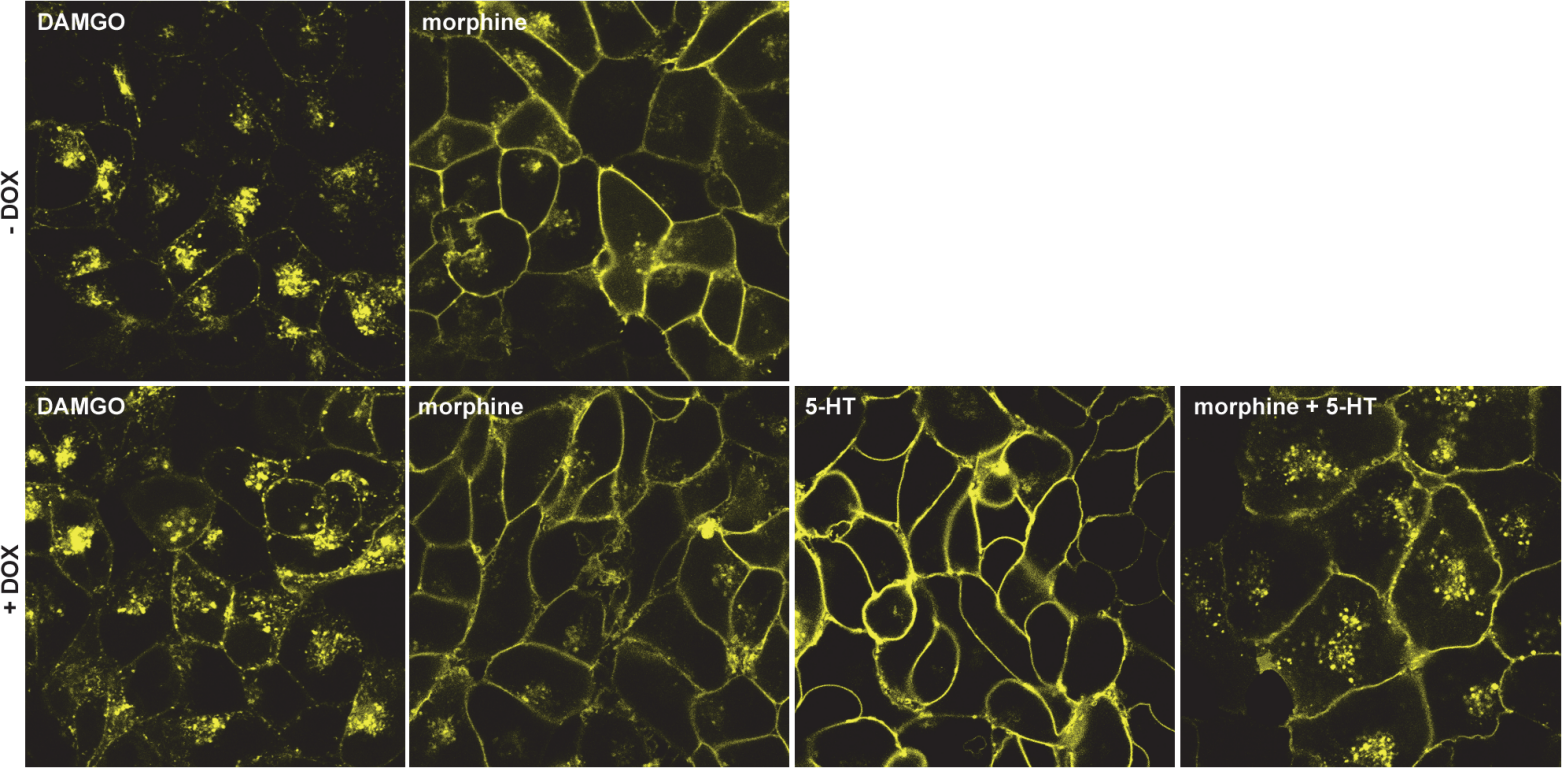
MOP-YFP receptor endocytosis studies in living cells by confocal microscopy. **Upper panels:** Confocal microscopy images from Flp-In HEK293 cells permanently expressing MOP-YFP receptors without induction of c-myc-5-HT_2C_-Cerulean receptor expression. Cells were treated with either DAMGO (10 μM) (left panel) or morphine (10 μM) (right panel) for 30 minutes before picture acquisition. **Lower panels:** Images from cells treated with doxycycline (0.01 μg/ml) for 24 hours in order to express c-myc-5-HT_2C_-Cerulean receptors. Cells were treated with either DAMGO (10 μM), morphine (10 μM), 5-HT (10 μM) or morphine plus 5-HT for 30 minutes before picture acquisition.

Treatment of cells only expressing opioid receptors with the synthetic enkephalin analogue DAMGO for 30 minutes resulted in a redistribution of the YFP fluorescent signal to cytosolic compartments in the form of dots and punctuate aggregates (Fig 2), suggesting the occurrence of endocytosis upon this agonist treatment. On the contrary, MOP-YFP receptors hardly internalize when equivalent experiments were conducted with morphine (Fig 2). We recently described that MOP receptor internalization by morphine is facilitated by a mechanism depending on Gα_q/11_ coupling and PKC activation when serotonin 5-HT_2A_ receptors expressed in the same cells were concomitantly activated [4]. Serotonin 5-HT_2C_ receptor exhibits a high structural homology to 5-HT_2A_ and as a result both receptors initiate basically the same cell signalling cascades upon activation [11]; consequently, it could be anticipated a potentiation of MOP receptor endocytosis promoted by morphine when 5-HT_2C_ receptor is concomitantly stimulated. Indeed, when c-myc-5-HT_2C_-Cerulean receptors were induced to express in the double-stable cell line, treatment of cells with morphine in combination with serotonin for 30 minutes resulted in the internalization of the opioid receptor at an apparent similar extent to that observed for equivalent assays performed with DAMGO (Fig 2). Conversely, either morphine or serotonin failed to evoke MOP-YFP receptor endocytosis when used separately (Fig 2).

**Fig 3:**
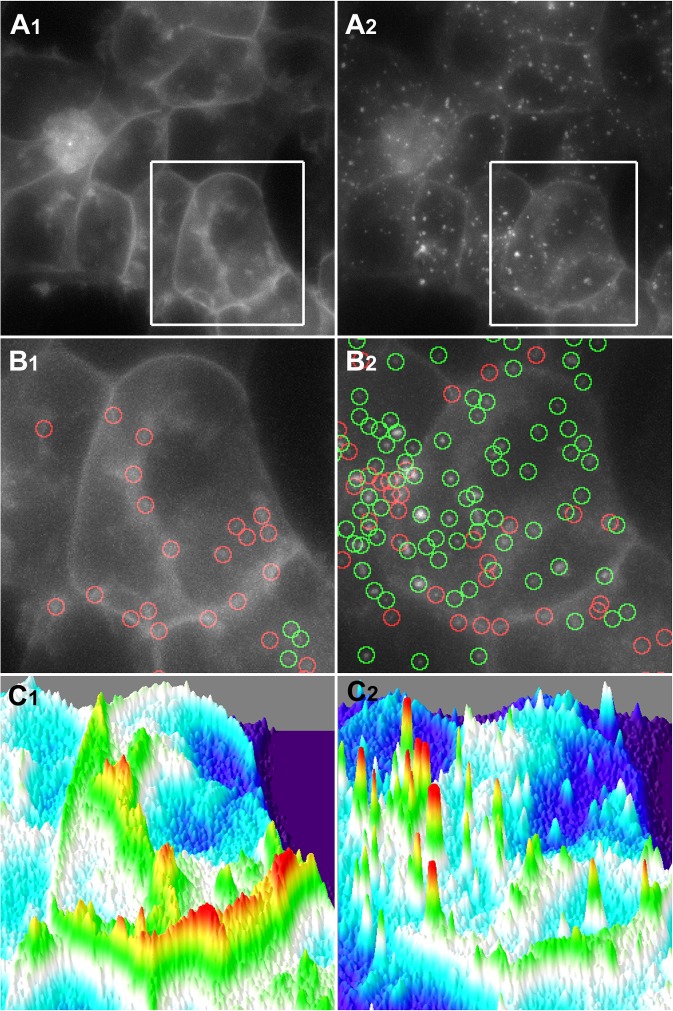
Selective detection of endosomes in fluorescence microscopy images from living cells. **A1,2:** Images corresponding to the same microscope field acquired at time = 0 (A1) and at time = 10 (A2) minutes upon DAMGO (10 μM) treatment. **B1,2:** Detailed images from cells indicated by the white frame in panels A. Green circles correspond to those local maxima (red circles) identified as endosomes by the algorithm. **C1,2:** 3D representation of the intensity of fluorescence signal from cells shown in panels B. Fluorescence corresponding to the plasma membrane follows a crest silhouette (C1) whereas endosome signal distribute as a spiky pattern (C2).

Time-lapse images obtained by epifluorescence microscopy from cells expressing both receptors and treated for 15 minutes with DAMGO, sufentanyl, morphine plus serotonin or morphine all of them at maximal efficacious doses (S1 Video) confirmed the incapability of morphine to induce MOP-YFP receptor internalization. Additionally, detailed observations of these movies revealed that the endocytosis promoted by morphine plus serotonin was clearly differentiated from those ones resulted for DAMGO and sufentanyl in both temporal and quantitative terms. This observation prompted us to conceive an analytical method (i.e. the Q-Endosomes algorithm) to evaluate the appearance of endosomes as a result of receptor activation by agonists in images obtained from real time experiments. Based in diverse analytical approaches previously described to detect endosomes in images obtained by fluorescence microscopy, particularly those ones used for single particle tracking (SPT) studies [12–14], we have developed a computer algorithm that identifies multiple particles corresponding to endosomes while discriminates these punctuate particles from the fluorescent signal corresponding to the cell plasma membrane. At difference of more comprehensive SPT methods, our interest at this point is focussed in the identification and discernment of endosomes as long as they become visible at the different time points upon receptor agonist stimulation rather than being concerned in the dynamics of endocytic vesicles through intracellular compartments once they were formed. Therefore and taking into account this premise, the Q-Endosomes algorithm was devised to detect fluorescent objects that fulfilled the following criteria: firstly, the particle was a local maxima; secondly, its intensity value was higher than the local background, and thirdly, it fitted adequately (R>0.75) a 2D-Gaussian function with a sigma value equivalent to 2.30 pixels (corresponding to 0.29 μm; see S1 Supplemental Data for a more detailed description of the algorithm development). To graphically illustrate the process, Fig 3 shows a particular microscopy field before (column 1, time = 0 minutes) and after (column 2, time = 10 minutes) treatment with DAMGO (10μM). Detailed images of an individual cell (Fig 3B) display those local maxima initially detected by the algorithm (red circles) and which of them are selected as endosomes (green circles). Basically, by introducing the Gaussian fitting, this image analysis method is able to differentiate the fluorescent signal following a crest contour as observed in the plasma membrane (Fig 3, C1), from that one closest to a spiky pattern of distribution typical of endosomes (Fig 3, C2). In order to evaluate the accuracy of the method, which we estimated to be above 90% (see S1 Supplemental Data), we tested the algorithm on simulated data by adding a known number of randomly distributed endosomes to a baseline image.

**Fig 4:**
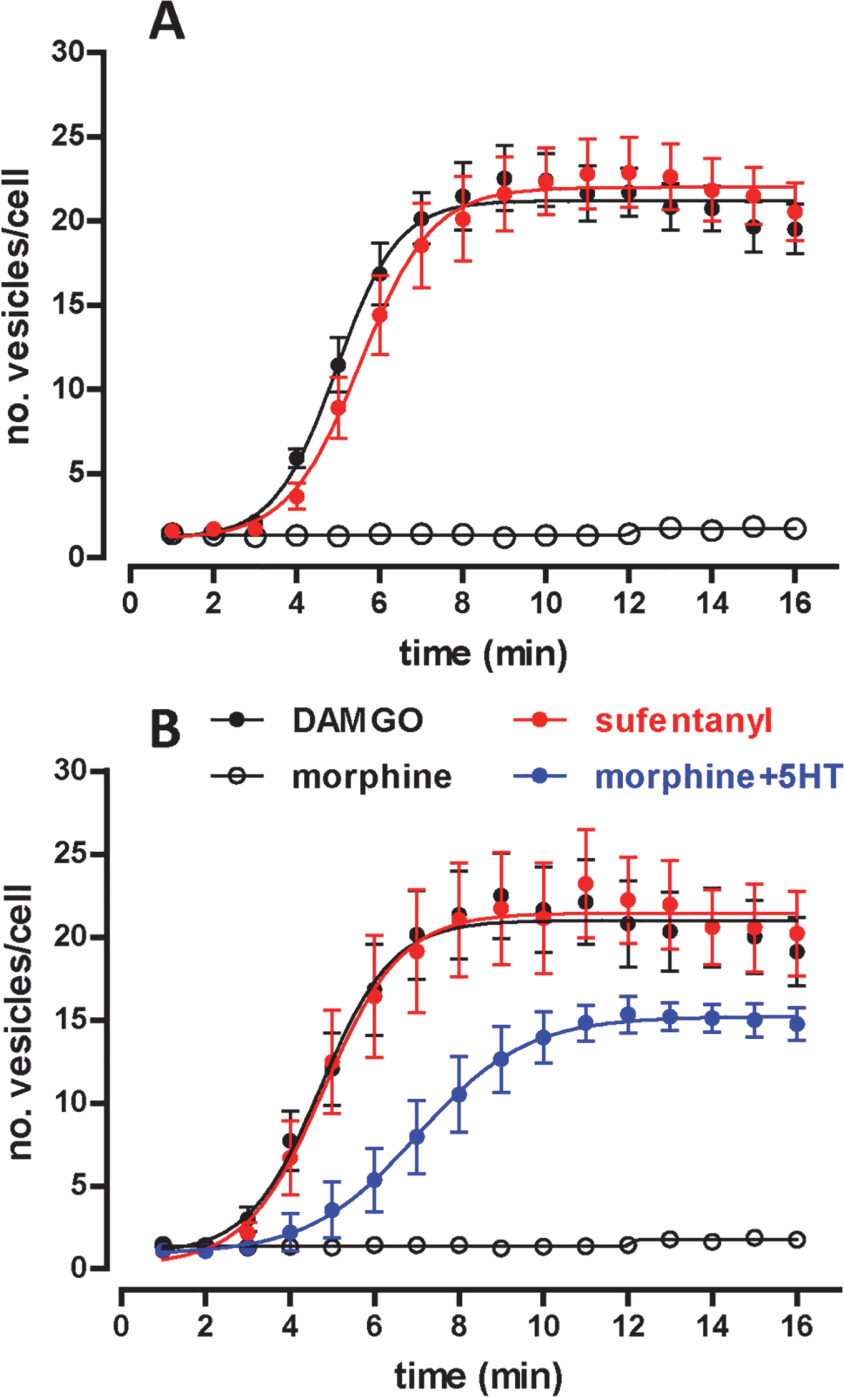
Time course of the generation of endocytic vesicles containing MOP-YFP receptors upon treatment with different agonist compounds. **A.** Graph shows the quantitative data obtained after image analysis of cells expressing MOP-YFP receptors and treated with DAMGO (10 μM) (black circles), sufentanyl (1 μM) (red circles) or morphine (10 μM) (open circles) for 15 minutes. Each point represents the mean ± SEM of at least three independent assays. **B.** Data corresponding to equivalent experiments from cells co-expressing MOP-YFP and c-myc-5-HT_2C_-Cerulean receptors and treated with DAMGO (10 μM) (black circles), sufentanyl (1 μM) (red circles), morphine (10 μM) (open circles) or morphine (10 μM) plus 5-HT (10 μM) (blue circles) for 15 minutes. Each point represents the mean ± SEM of at least three independent assays.

Graphical representations of the number of fluorescent spots generated at different time points by activation of MOP-YFP receptors with several opioid agonists resulted in a data distribution fitting to a sigmoidal function, as shown by representative results in Fig 4.

By this new approach, the kinetics of this process can be precisely described in terms of the three characteristic parameters of a sigmoid curve, i.e., the maximal effect (Emax) in this case the maximum number of spots detected corresponding to internalizing vesicles or endosomes per cell, the time to reach the 50% of that maximal response defined as half time (t_1/2_) and the steepness of the curve (slope). Tables 1 and 2 summarize the values corresponding to these parameters from independent experiments performed in cells expressing MOP-YFP receptors alone and cells expressing c-myc-5-HT_2C_-Cerulean and MOP-YFP receptors simultaneously. Cells only expressing MOP-YFP receptors showed a robust receptor internalization when they were treated for 15 minutes with DAMGO (10 μM), sufentanyl (1μM) and methadone (10 μM) with no significant difference among them in relation to t_1/2_, maximum number of vesicles per cell or curve slope (one-way ANOVA, P>0.05). In all the three cases the maximal effect (between 20 and 27 vesicles per cell) was reached in about 10 minutes with a half time of 5 minutes approximately (Table 1). Conversely, neither morphine, oxycodone nor buprenorphine generated significant endocytosis of MOP-YFP receptors upon 15 minutes of treatment at 10 μM. Equivalent experiments were conducted in cells pre-treated with doxycycline to induce the expression of c-myc-5-HT_2C_-Cerulean receptors (Table 2). In this later case, either kinetics parameters (t_1/2_ and curve slope) as maximum response (Emax) observed for DAMGO, sufentanyl, methadone and morphine were not significantly different to the ones obtained with the same compounds in cells only expressing MOP-YFP receptors (t-test, P>0.05). As we mentioned above when describing the characterisation of the double-stable cell line by confocal microscopy, the co-activation of c-myc-5-HT_2C_-Cerulean with serotonin (5-HT) facilitates the endocytosis of MOP-YFP receptors by morphine at an apparent extent than DAMGO. However, the quantitative analysis of equivalent experiments conducted in real time by epifluorescence microscopy with living cells revealed that both processes, i.e., MOP-YFP endocytosis induced by either DAMGO or morphine plus 5-HT, were significantly different in terms of kinetics (t_1/2_) and efficacy (Emax) (one-way ANOVA P<0.005) (Fig 4 and Table 2). That capacity to distinguish among different endocytosis events depending on the agonist compounds used to initiate the process demonstrates the selectivity of this new method. In a similar way, experiments using other agonists for 5-HT_2C_ receptors known to be partially efficacious in relation to 5-HT in more conventional functional assays were conducted to confirm the robustness of this technique. Thus, treatment of cells with either DOI (10 μM) or WAY161503 (1μM) in conjunction with morphine resulted in MOP-YFP receptor endocytosis kinetically equivalent to the one observed when 5-HT was used as agonist (Table 2); on the contrary, both DOI and WAY161503 generated a significant lower number of vesicles per cell than 5-HT (t-test P<0.05) (Table 2). Therefore, these results indicate that the differences in intrinsic activity among 5-HT, DOI and WAY161503 observed previously in both intracellular calcium mobilisation assays and endocytosis induction of 5-HT_2C_-GFP receptors transiently expressed in HEK293 cells [15] are also discernible with this approach when evaluating the effect of these agonists to facilitate MOP-YFP endocytosis by morphine. Intriguingly, that facilitation of MOP-YFP internalization observed in the case of morphine was not emulated by other opioid agonist such as oxycodone or buprenorphine also incompetent by themselves to promote MOP receptor endocytosis.

**Table 1:** Kinetic parameters obtained from endocytosis assays performed with cells heterologously expressing MOP-YFP receptors.

	t_1/2_(min.)	Emax (no. vesicles/cell)	Slope	n
**DAMGO**	5.02 ± 0.20	21.29 ± 1.49	0.59 ± 0.05	5
**Sufentanyl**	5.62 ± 0.31	23.44 ± 2.60	0.47 ± 0.04	5
**Methadone**	5.69 ± 0.36	26.16 ± 2.34	0.39 ± 0.05	3
**Morphine**	n.d.	n.d.	n.d.	3
**Oxycodone**	n.d.	n.d.	n.d.	3
**Buprenorphine**	n.d.	n.d.	n.d.	3

Cells were treated with DAMGO (10 μM), sufentanyl (1 μM), methadone (10 μM), morphine (10 μM), oxycodone (10 μM) or buprenorphine (10 μM) for 15 minutes. Data indicate mean ± SEM of (n) independent experiments. t_1/2_: time to reach the 50% of the maximal response (minutes). Emax: maximal response (number of vesicles per cell). n.d.: not detected.

**Table 2:** Kinetic parameters obtained from endocytosis assays performed with cells heterologously co-expressing MOP-YFP and c-myc-5-HT2C-Cerulean receptors.

	t_1/2_ (min.)	Emax (no. vesicles/cell)	Slope	n
**DAMGO**	4.82 ± 0.24	21.00 ± 2.41	0.53 ± 0.01	5
**Sufentanyl**	4.74 ± 0.47	19.69 ± 3.26	0.74 ± 0.12	4
**Methadone**	5.92 ± 0.61	29.28 ± 2.56	0.43 ± 0.08	3
**Morphine**	n.d.	n.d.	n.d.	3
**Morphine + 5-HT**	7.38 ± 0.70	14.72 ± 0.27	0.46 ± 0.04	5
**Morphine + DOI**	8.47 ± 1.09	8.18 ± 1.31	0.47 ± 0.13	5
**Morphine + WAY161503**	8.86 ± 0.93	9.08 ± 1.88	0.31 ± 0.04	4
**Oxycodone + 5-HT**	n.d.	n.d.	n.d.	3
**Buprenorphine + 5-HT**	n.d.	n.d.	n.d.	3

Cells were treated with DAMGO (10 μM), sufentanyl (1 μM), methadone (10 μM), morphine (10 μM), morphine (10 μM) plus 5-HT (10 μM), morphine (10 μM) plus DOI (10 μM), morphine (10 μM) plus WAY161503 (1 μM), oxycodone (10 μM) plus 5-HT (10 μM) or buprenorphine (10 μM) plus 5-HT (10 μM). Data indicate mean ± SEM of (n) independent experiments. t_1/2_: time to reach the 50% of the maximal response (minutes). Emax: maximal response (number of vesicles per cell). n.d.: not detected.

**Fig 5:**
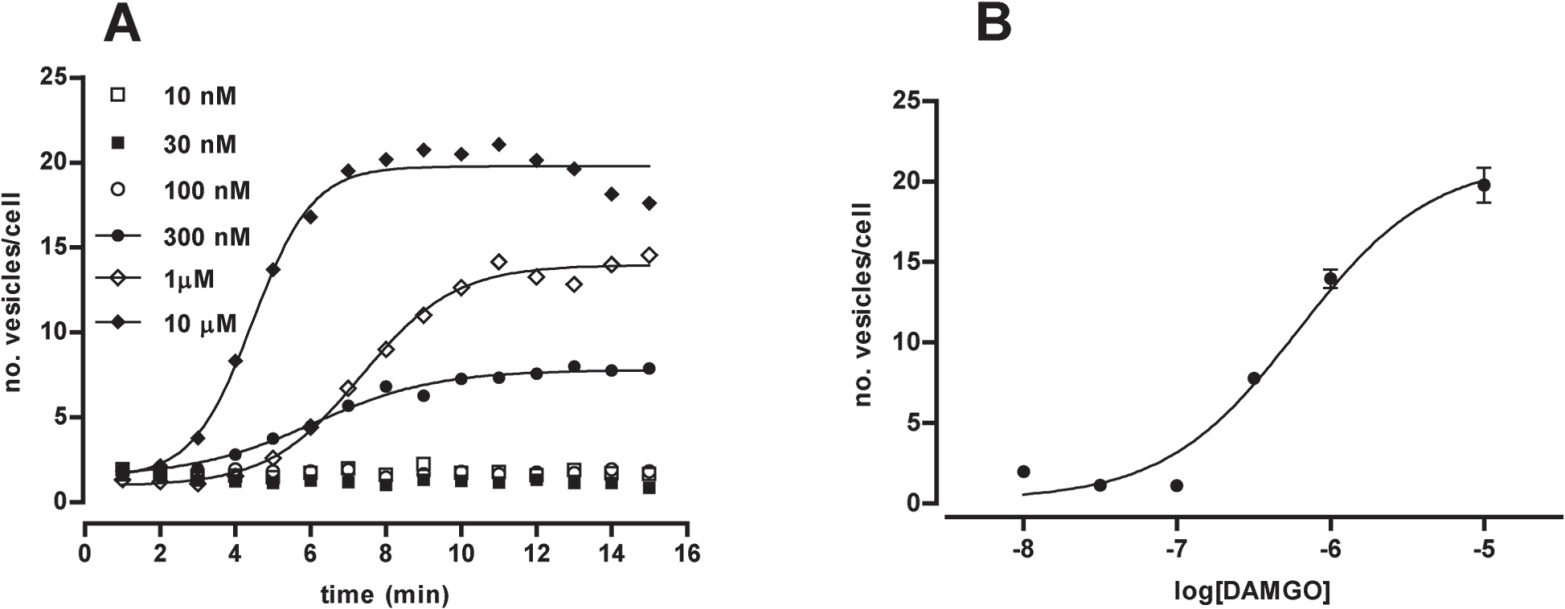
Effect of different doses of DAMGO on the time-course generation of endocytic vesicles containing MOP-YFP receptors. **A.** Data graph show representative results from time-course MOP-YFP receptor endocytosis experiments performed with DAMGO at different concentrations (see symbol legends). **B.** Dose-response curve obtained after non-linear analysis of data corresponding to the maximal effect in terms of number of vesicles per cells generated by different concentrations of DAMGO. Each point represents the mean ± SEM of three independent experiments.

According to the mostly established and accepted classical theories in receptor pharmacology, the receptor concept is conceived as a system where a particular input in chemical form (agonist compound) results in a detectable output in form of biological response. That amount of response or effect should be measurable by analytical means and the capacity of any given compound to produce it is known as efficacy. Then, different drugs may have varying capacities to initiate a response through a particular receptor and there is not an absolute scale for efficacy but rather it is a dealt with in relative terms, i.e. the ratio of the efficacy of two different drugs on a particular biological system [16]. To further validate our methodology to evaluate receptor endocytosis as a functional pharmacological assay, MOP-YFP receptor endocytosis experiments in real time were performed by using different concentrations of the agonist DAMGO. Fig 5A displays the different kinetic curves obtained with different doses of DAMGO; thus, either 10 nM, 30 nM or 100 nM produced undetectable responses in terms of endocytic vesicles along 15 minutes of image acquisition. Per contra, higher amounts of DAMGO, i.e., 300 nM, 1 μM and 10μM, generated the formation of endocytic vesicles in a dose dependent manner (Fig 5A). When the maximal response obtained with different DAMGO concentrations from three independent experiments were plotted on a semi logarithmic graph, a sigmoidal dose-response curve corresponding to the effect of DAMGO on MOP-YFP endocytosis was obtained (Fig 5B) resulting in a potency (pEC50) of 6.21 ± 0.15. This value is comparable to the one previously obtained with the same agonist in [^35^S]GTPγS binding assays performed with membranes from Flp-In HEK 293 cells permanently expressing MOP-YFP receptors (7.58 ± 0.09) [4].

**Fig 6:**
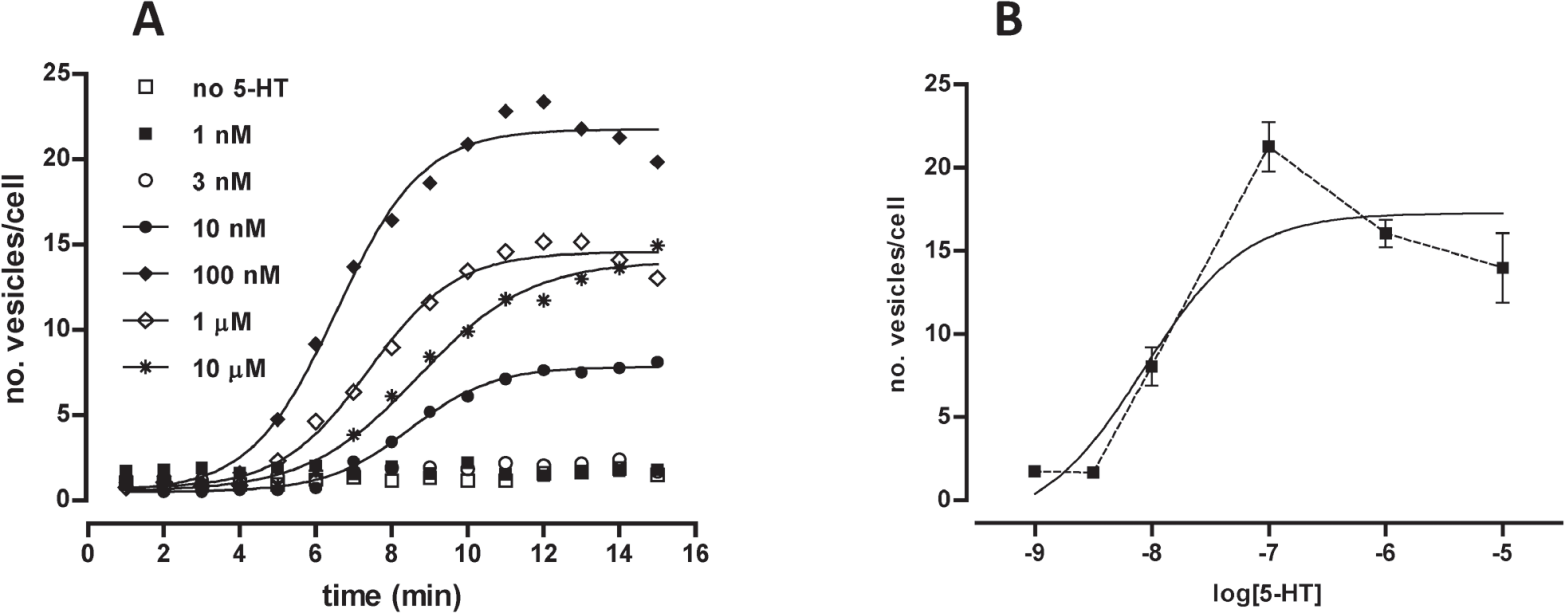
Effect of different doses of serotonin (5-HT) on the facilitation of the endocytosis of MOP-YFP receptors by morphine. **A.** Data graph display representative results from time-course MOP-YFP receptor endocytosis experiments conducted by co-treatment with morphine at 10 μM plus 5-HT at different concentrations (see symbol legends). **B.** Dose-response curve (continuous line) obtained after non-linear analysis of the maximal effect (number of vesicles per cell) generated by morphine (10 μM) plus different concentrations of 5-HT. Each point represents the mean ± SEM of three independent experiments. Dotted line display the characteristic bell shaped profile observed when connecting these points in an increasing dose-dependent manner.

In a similar manner, if the facilitation of MOP-YFP endocytosis by morphine observed with serotonin is mediated by the activation of c-myc-5-HT_2C_-Cerulean receptors, a dose depending response should be anticipated when assessing different concentrations of serotonin. Results from three independent experiments demonstrating this assumption are shown in Fig 6; briefly, lowest doses of serotonin (1nM and 10 nM) produced no facilitation of MOP-YFP endocytosis by morphine whereas higher doses (10 nM, 100 nM, 1 μM and 10 μM) resulted in the appearance of endocytic vesicles in a time dependent manner (Fig 6A). Unexpectedly, the highest and fastest effect was observed with serotonin at 100 nM rather than with more concentrated doses such as 1 μM or 10 μM, leading to a bell shaped dose-response curve when these results were plotted in a semi logarithm graph (Fig 6B). Non-linear regression of these data points fitted to a sigmoid dose-response function resulting in a potency (pEC50) of serotonin to that facilitation effect of 8.07 ± 0.52 (Fig 6B). This value is in accordance with the potency of serotonin to mobilize intracellular calcium stores through 5-HT_2C_-GFP receptors transiently expressed in HEK293 cells (1.80 ± 0.7 nM) [15]. Although the elucidation of the underlying molecular mechanisms responsible of this bell shaped profile is out of the scope of the present work, future investigations will be considered to explore the possible causes of such atypical response. Two main hypothesis could be regarded as speculative explanations: one of them is the coupling of 5-HT_2C_ receptors to different signalling pathways depending on the serotonin concentration acting in the system, and the other one is the presence of additional serotonin receptor subtypes endogenously expressed in HEK 293 cells presenting an inhibitory effect on the facilitation of c-myc-5-HT_2C_-Cerulean receptors at high serotonin concentrations.

**Fig 7:**
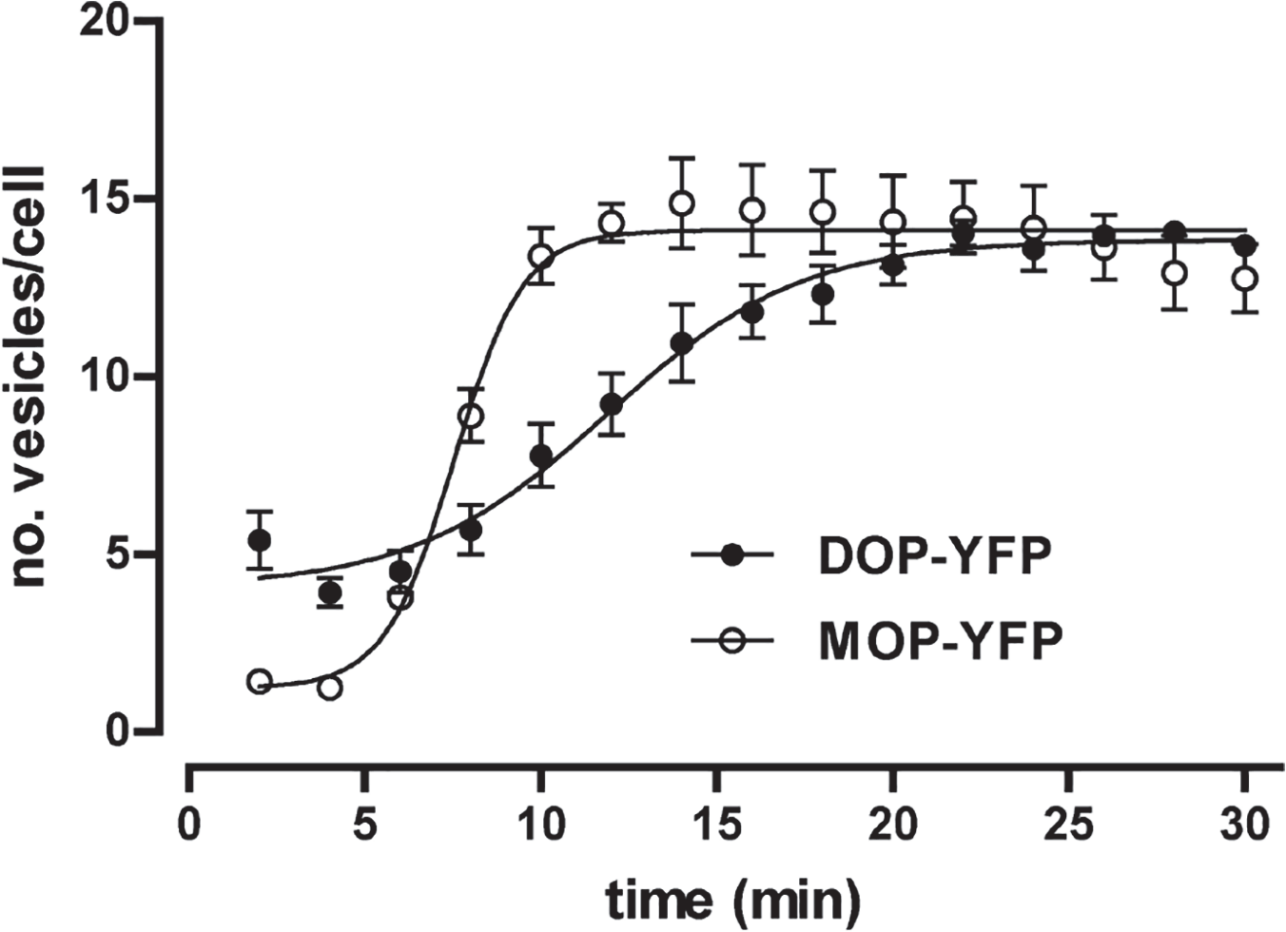
Time course of the generation of endocytic vesicles containing either DOP-YFP or MOP-YFP receptors transiently expressed in Flp-In T-REx HEK293 cells upon agonist treatment. Time-course curves showing the generation of endocytic vesicles obtained from images of cells expressing DOP-YFP or MOP-YFP receptors upon treatment with DTLET (10 μM) and DAMGO (10 μM) respectively.

The applicability of this method to a different GPCR was tested by using delta opioid receptor (DOP) tagged to YFP at the carboxi terminus since earlier investigations conducted in HEK 293 cells heterologously expressing either MOP or DOP receptors already described their endocytosis as a consequence of binding to opioid agonists [17]. Consequently, DOP-YFP receptors were transiently expressed in parental Flp-In HEK 293 cells to be submitted to agonist treatment in real time fluorescence microscopy assays. Similarly as observed for MOP-YFP receptors permanently expressed in Flp-In HEK 293 cells, DOP-YFP receptors clearly internalized upon treatment with the enkephalin analogue DTLET ([D-Thr2-Leu5]Enkephalin-Thr) (S2 Video). Results obtained from three independent experiments are shown in Fig 7. Briefly, treatment of cells with DTLET (10 μM) resulted in a time-dependent increase of the number of endocytic vesicles following a sigmoid curve with the next kinetic parameters: t_1/2_ = 11.90 ± 0.61 minutes, Emax = 13.84 ± 0.30 vesicles/cell and Slope = 0.16 ± 0.03 (n = 3). These data indicate that DOP-YFP receptor endocytosis process is appreciably slower than the one observed for MOP-YFP receptor in DAMGO treatment assays (Table 1). To control this issue in the same expression system, MOP-YFP receptors were transiently expressed in Flp-In HEK 293 cells to be treated with DAMGO at 10 μM for 30 minutes. Fig 7 displays the kinetic curve obtained from three independent experiments that resulted in the following values confirming the differences between MOP-YFP and DOP-YFP receptors: t_1/2_ = 7.54 ± 0.29 minutes, Emax = 14.11 ± 0.28 vesicles/cell and Slope = 0.44 ± 0.11 (n = 3). Another difference between MOP-YFP and DOP-YFP endocystosis processes, also discernible when both kinetic curves are plotted in the same graph, is the slight constitutive endocytosis of DOP-YFP detectable in basal conditions in absence of agonist stimulation (Fig 7).

**Fig 8:**
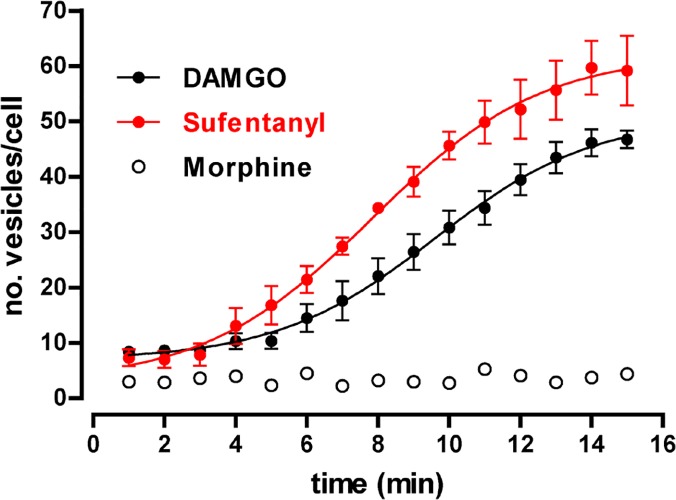
Time course of the generation of endocytic vesicles containing MOP-YFP receptors transiently expressed in HeLa cells upon treatment with different agonists. Time-course curves showing the generation of endocytic vesicles after treatment with DAMGO (10 μM), sufentanyl (1 μM) or morphine (10 μM) in HeLa cells expressing MOP-YFP receptors.

Trafficking of GPCRs is regulated by a vast variety of cellular interacting proteins [1, 18]. Therefore, and similarly as described for other functional responses, for any given receptor type different cellular hosts should have distinctive efficiencies of coupling to effectors that determines, for instance, the characteristics of endocytosis processes. To further explore on this circumstance by the analytical approach described herein, MOP-YFP receptors were transiently expressed in HeLa cells and endocytosis experiments were conducted in real time by fluorescence microscopy. Fig 8 compiles results obtained from three independent assays using DAMGO, sufentanyl and morphine as agonist drugs.

Comparatively as observed in Flp-In HEK 293 cells, morphine totally failed to induce any endocytosis of MOP-YFP receptors. Conversely, either DAMGO as sufentanyl caused a significant formation of internalizing vesicles in a time dependent manner (Fig 8) (S3 Video). Table 3 summarizes the kinetic parameters obtained from the analysis of DAMGO and sufentanyl curves; in both cases, the process was significantly slower than the equivalent one occurred in FLP-In HEK 293 cells (t-test P<0.05, when comparing either t_1/2_ or Slope). Another significant disparity between FLP-In HEK 293 and HeLa cell results was the maximal effect obtained for both opioid agonists in terms of number of vesicles detected per cell (Tables 1 and 3). The reason for this discrepancy may be due to the phenotypic differences between these cell lines since HeLa cells, although thinner than HEK 293 (≈4 μM *versus* ≈10 μM respectively), are noticeably larger than HEK 293 (compare bar scale and cell size between images from S2 Video and S3 Video). Altogether, these results confirm what is accepted for other experimental models to evaluate functionality in receptor pharmacology studies, that is, efficacy as a magnitude rather than being an absolute value it should be considered in relative terms when comparing two different drugs on a particular biological system.

**Table 3:** Kinetic parameters obtained from endocytosis assays performed with HeLa cells transiently expressing MOP-YFP receptors.

	t_1/2_ (min.)	Emax (no. vesicles/cell)	Slope	n
**DAMGO**	9.60 ± 0.48	50.85 ± 3.89	0.19 ± 0.04	3
**Sufentanyl**	7.83 ± 0.45	62.25 ± 3.95	0.18 ± 0.04	
**Morphine**	n.d.	n.d.	n.d.	3

Cells were treated with DAMGO (10 μM), sufentanyl (1 μM) or morphine (10 μM) for 15 minutes. Data indicate mean ± SEM of (n) independent experiments. t_1/2_: time to reach the 50% of the maximal response (minutes). Emax: maximal response (number of vesicles per cell). n.d.: not detected.

Other methodologies previously described to quantitative assess GPCRs endocytosis are also based on mathematical algorithms to detect fluorescent aggregates in images obtained by microscopy. This is the case of the ArrayScan technology initially developed as a high-content screening system [19–20] to be used with cells heterologously expressing receptor-fluorescent protein fusion conjugates such as parathyroid hormone receptor PTH-GFP or β_2_-GFP adrenoceptor, and lately validated with serotonin 5-HT_2C_-GFP [15], vasopressin V_1a_-GFP, proton sensing TDAG8-GFP and free fatty acid GPR120-GFP receptors [21]. Although this technology permits to conduct receptor functional studies comparable, at some extent, to other more conventional pharmacology assays [15], its main technical limitation lies in the inability to define plasma membrane and, thereby, to eliminate this background signal. Besides this lack of discrimination of the fluorescent signal originated at the plasma membrane, the major difference between ArrayScan algorithm and the new one described herein resides in the nature of the fluorescent object to be detected and therefore quantified. Thus, the former method considers as quantifiable ‘spots’ the accumulation of endocytic vesicles in larger structures close to the nuclear compartment also defined as perinuclear endocytic recycling (ERC) compartment [19–22], whereas the novel approach described in here is capable to identify individual vesicles from the commencement of the endocytosis process and at different cellular locations. This higher versatility confers to our method the temporal and operational resolution necessary to carry out functional experiments in real time. Previous investigations attempted to analyse the timing of receptor endocytosis finding out the onset and duration of the process [23]; these studies consisted in the examination in real time of the internalization of cAR1 receptors upon external stimulation by cAMP *in vivo* in *Dictyostelium discoideum* by single-molecule microscopy. However, although this is a very sensitive imaging technology, it requires some methodological requirements that makes its implementation difficult to perform, i.e., previous photobleaching of the sample or acquisition of stacks of 100 images with a time lag between images of 5 ms.

The validation of the procedure described in this report with different GPCRs as well as diverse cellular phenotypes further supports the applicability of this method to varied experimental situations. In fact, the only prerequisite for the use of this methodology is to have a level of receptor expression in the heterologous system that permits the detection of endosomes by fluorescence microscopy.

In conclusion and based on already described algorithms to detect cellular punctuate objects in single particle tracking studies, we have implemented an image analysis method to evaluate receptor endocytosis as a pharmacological response by fluorescence living cell microscopy. The functional and temporal resolution of this new approach is high enough to characterize the pharmacological profile of those chemical compounds used to initiate this biological process. In this sense, the contribution of experimental tools as the one described herein is essential to determine the intrinsic activity of potential drug candidates when considering endocytosis as a druggable target.

## Supporting Information

S1 Fig Characterization of endosomes from real microscopy images. **A,B:** 3D plots of a representative endosome before (A) and after (B) smoothing the image with a Gaussian filter of sigma = 1 pixel. *X* and *Y* axis units are pixels whereas *Z* axis corresponds to fluorescence intensity (a.f.u.). **C:** Box-and-Whisker plots of sigma values (pixels) of local maxima corresponding to endosomes (gray box) or to other non-endosome cellular structures, mainly plasma membrane (white box). **D:** Box-and-Whisker plots of correlation values corresponding to the fitting to a 2D-Gaussian function of sigma = 2.17 pixels obtained from endosomes (gray box) and non-endosome structures (white box).
(TIFF)

S2 Fig Flow-chart cartoon summarizing Q-Endosomes algorithm rationale. (TIFF)

S3 Fig Model to simulate endosome formation. **A:** Image from untreated cells obtained after Z-stack maximum projection. **B:** Thresholding of image A to separate those areas containing cells. **C:** Random distribution of simulated endosomes on these regions corresponding to cells. **D:** Merging of images A and C.
(TIF)

S4 Fig A: Accuracy of the Q-endosomes algorithm varying the radio of the region to be fitted from 2 to 7 pixels. The figure shows the ratio found between the number of detected and simulated endosomes, averaged across 10 independent simulations. Inset graph indicates the relative error between simulated and detected values. **B:** surface graph showing the relative error found between simulated and detected values from simulations performed by simultaneously modifying values corresponding to threshold above local background and correlation coefficient of the 2D-Gaussian function.
(TIFF)

S1 Q_Endosomes Matlab code corresponding to Q-Endosomes algorithm. (HTML)

S1 Supplemental Data (DOCX)

S1 Video Real time images obtained from living cells by fluorescence microscopy. The fluorescent signal corresponds to MOP-YFP receptors and the final image is the result of the Z-stack maximal projection. Cells were treated for 15 minutes with the following agonists: DAMGO (10 µM), morphine (10 µM), sufentanyl (1 µM) and morphine (10 µM) plus serotonin (10 µM). Images were acquired every 1 minute. Clock in the top left corner of each panel indicates real time in minutes.
(MOV)

S2 Video Real time images from Flp-In HEK 203 cells transiently expressing DOP-YFP receptors. Cells were treated with 10 µM of DTLET for 30 minutes. Images were acquired every 2 minutes. Clock in the top left corner indicates real elapsed time in minutes.
(MOV)

S3 Video Real time images from HeLa cells transiently expressing MOP-YFP receptors. Cells were treated with 10 µM of DAMGO for 15 minutes and images were acquired every 1 minute. Clock in the top left corner indicates real elapsed time in minutes.
(MOV)

## References

[1] Hanyaloglu AC, von Zastrow M (2008) Regulation of GPCRs by endocytic membrane trafficking and its potential implications. Annu Rev Pharmacol Toxicol 48: 537–568. 10.1146/annurev.pharmtox.48.113006.094830 18184106

[2] Irannejad R, Tomshine JC, Tomshine JR, Chevalier M, Mahoney JP, et al (2013) Conformational biosensors reveal GPCR signalling from endosomes. Nature 495: 534–538. 10.1038/nature12000 23515162 PMC3835555

[3] Tsien RY (1998) The green fluorescent protein. Annu Rev Biochem 67: 509–544. 9759496 10.1146/annurev.biochem.67.1.509

[4] Lopez-Gimenez JF, Vilaro MT, Milligan G (2008) Morphine desensitization, internalization, and down-regulation of the mu opioid receptor is facilitated by serotonin 5-hydroxytryptamine2A receptor coactivation. Mol Pharmacol 74: 1278–1291. 10.1124/mol.108.048272 18703670

[5] Urban JD, Clarke WP, von Zastrow M, Nichols DE, Kobilka B, et al (2007) Functional selectivity and classical concepts of quantitative pharmacology. J Pharmacol Exp Ther 320: 1–13. 16803859 10.1124/jpet.106.104463

[6] Raehal KM, Schmid CL, Groer CE, Bohn LM (2011) Functional selectivity at the mu-opioid receptor: implications for understanding opioid analgesia and tolerance. Pharmacol Rev 63: 1001–1019. 10.1124/pr.111.004598 21873412 PMC3186080

[7] Williams JT, Ingram SL, Henderson G, Chavkin C, von Zastrow M, et al (2013) Regulation of mu-opioid receptors: desensitization, phosphorylation, internalization, and tolerance. Pharmacol Rev 65: 223–254. 10.1124/pr.112.005942 23321159 PMC3565916

[8] Whistler JL (2012) Examining the role of mu opioid receptor endocytosis in the beneficial and side-effects of prolonged opioid use: from a symposium on new concepts in mu-opioid pharmacology. Drug Alcohol Depend 121: 189–204. 10.1016/j.drugalcdep.2011.10.031 22226706 PMC4224378

[9] Lopez-Gimenez JF, Milligan G (2010) Opioid regulation of mu receptor internalisation: relevance to the development of tolerance and dependence. CNS Neurol Disord Drug Targets 9: 616–626. 20632966 10.2174/187152710793361522

[10] Berger AC, Whistler JL (2010) How to design an opioid drug that causes reduced tolerance and dependence. Ann Neurol 67: 559–569. 10.1002/ana.22002 20437553 PMC2943346

[11] Leysen JE (2004) 5-HT2 receptors. Curr Drug Targets CNS Neurol Disord 3: 11–26. 14965241 10.2174/1568007043482598

[12] Cheezum MK, Walker WF, Guilford WH (2001) Quantitative comparison of algorithms for tracking single fluorescent particles. Biophys J 81: 2378–2388. 11566807 10.1016/S0006-3495(01)75884-5 PMC1301708

[13] Jaqaman K, Loerke D, Mettlen M, Kuwata H, Grinstein S, et al (2008) Robust single-particle tracking in live-cell time-lapse sequences. Nat Methods 5: 695–702. 10.1038/nmeth.1237 18641657 PMC2747604

[14] Ruthardt N, Lamb DC, Brauchle C (2011) Single-particle tracking as a quantitative microscopy-based approach to unravel cell entry mechanisms of viruses and pharmaceutical nanoparticles. Mol Ther 19: 1199–1211. 10.1038/mt.2011.102 21654634 PMC3129551

[15] Schlag BD, Lou Z, Fennell M, Dunlop J (2004) Ligand dependency of 5-hydroxytryptamine 2C receptor internalization. J Pharmacol Exp Ther 310: 865–870. 15113845 10.1124/jpet.104.067306

[16] Stephenson RP (1956) A modification of receptor theory. Br J Pharmacol Chemother 11: 379–393. 13383117 10.1111/j.1476-5381.1956.tb00006.x PMC1510558

[17] Keith DE, Murray SR, Zaki PA, Chu PC, Lissin DV, et al (1996) Morphine activates opioid receptors without causing their rapid internalization. J Biol Chem 271: 19021–19024. 8702570 10.1074/jbc.271.32.19021

[18] Magalhaes AC, Dunn H, Ferguson SS (2012) Regulation of GPCR activity, trafficking and localization by GPCR-interacting proteins. Br J Pharmacol 165: 1717–1736. 10.1111/j.1476-5381.2011.01552.x 21699508 PMC3372825

[19] Ghosh RN, Chen YT, DeBiasio R, DeBiasio RL, Conway BR, et al (2000) Cell-based, high-content screen for receptor internalization, recycling and intracellular trafficking. Biotechniques 29: 170–175. 10907092 10.2144/00291pf01

[20] Conway BR, Minor LK, Xu JZ, Gunnet JW, DeBiasio R, et al (1999) Quantification of G-Protein Coupled Receptor Internatilization Using G-Protein Coupled Receptor-Green Fluorescent Protein Conjugates with the ArrayScantrade mark High-Content Screening System. J Biomol Screen 4: 75–86. 10838415 10.1177/108705719900400207

[21] Fukunaga S, Setoguchi S, Hirasawa A, Tsujimoto G (2006) Monitoring ligand-mediated internalization of G protein-coupled receptor as a novel pharmacological approach. Life Sci 80: 17–23. 16978657 10.1016/j.lfs.2006.08.022

[22] Haasen D, Schnapp A, Valler MJ, Heilker R (2006) G protein-coupled receptor internalization assays in the high-content screening format. Methods Enzymol 414: 121–139. 17110190 10.1016/S0076-6879(06)14008-2

[23] Serge A, de Keijzer S, Van Hemert F, Hickman MR, Hereld D, et al (2011) Quantification of GPCR internalization by single-molecule microscopy in living cells. Integr Biol (Camb) 3: 675–683. 10.1039/c0ib00121j 21541374

[24] Ehrhardt C, Schmolke M, Matzke A, Knoblauch A, Will C, et al (2006) Polyethylenimine, a cost-effective transfection reagent. Signal Transduction 6: 179–184.

[25] Ward RJ, Alvarez-Curto E, Milligan G (2011) Using the Flp-In T-Rex system to regulate GPCR expression. Methods Mol Biol 746: 21–37. 10.1007/978-1-61779-126-0_2 21607850

