# Supplementary figures and images for: Endocytosis as a Biological Response in Receptor Pharmacology: Evaluation by Fluorescence Microscopy
Source: PLoS One. 2015 Apr 7;10(4):e0122604. doi: 10.1371/journal.pone.0122604 (PMC4388511; doi:10.1371/journal.pone.0122604)

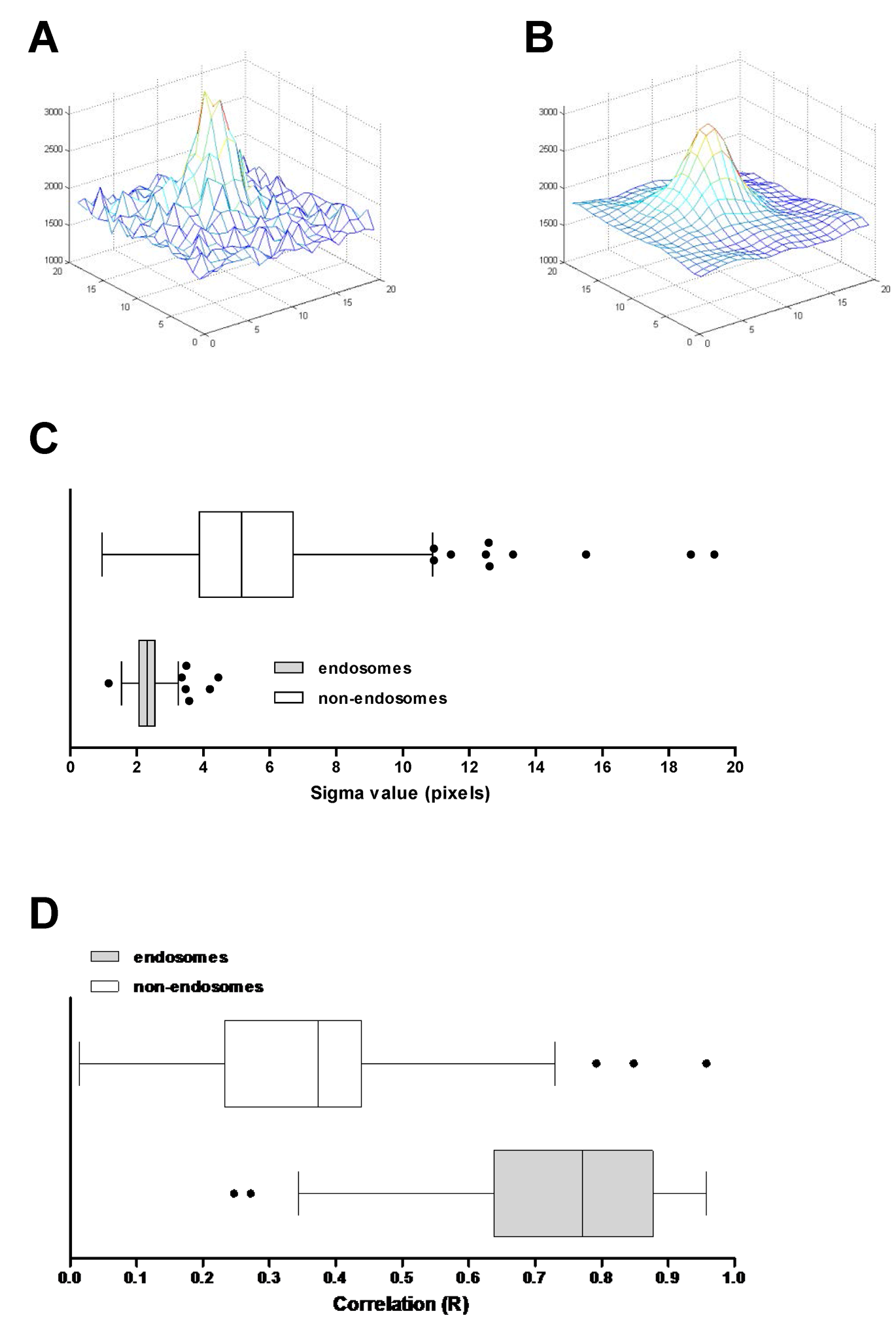

Supplement: S1 Fig — A,B: 3D plots of a representative endosome before (A) and after (B) smoothing the image with a Gaussian filter of sigma = 1 pixel. X and Y axis units are pixels whereas Z axis corresponds to fluorescence intensity (a.f.u.). C: Box-and-Whisker plots of sigma values (pixels) of local maxima corresponding to endosomes (gray box) or to other non-endosome cellular structures, mainly plasma membrane (white box). D: Box-and-Whisker plots of correlation values corresponding to the fitting to a 2D-Gaussian function of sigma = 2.17 pixels obtained from endosomes (gray box) and non-endosome structures (white box). (TIFF) [file pone.0122604.s001.tiff]

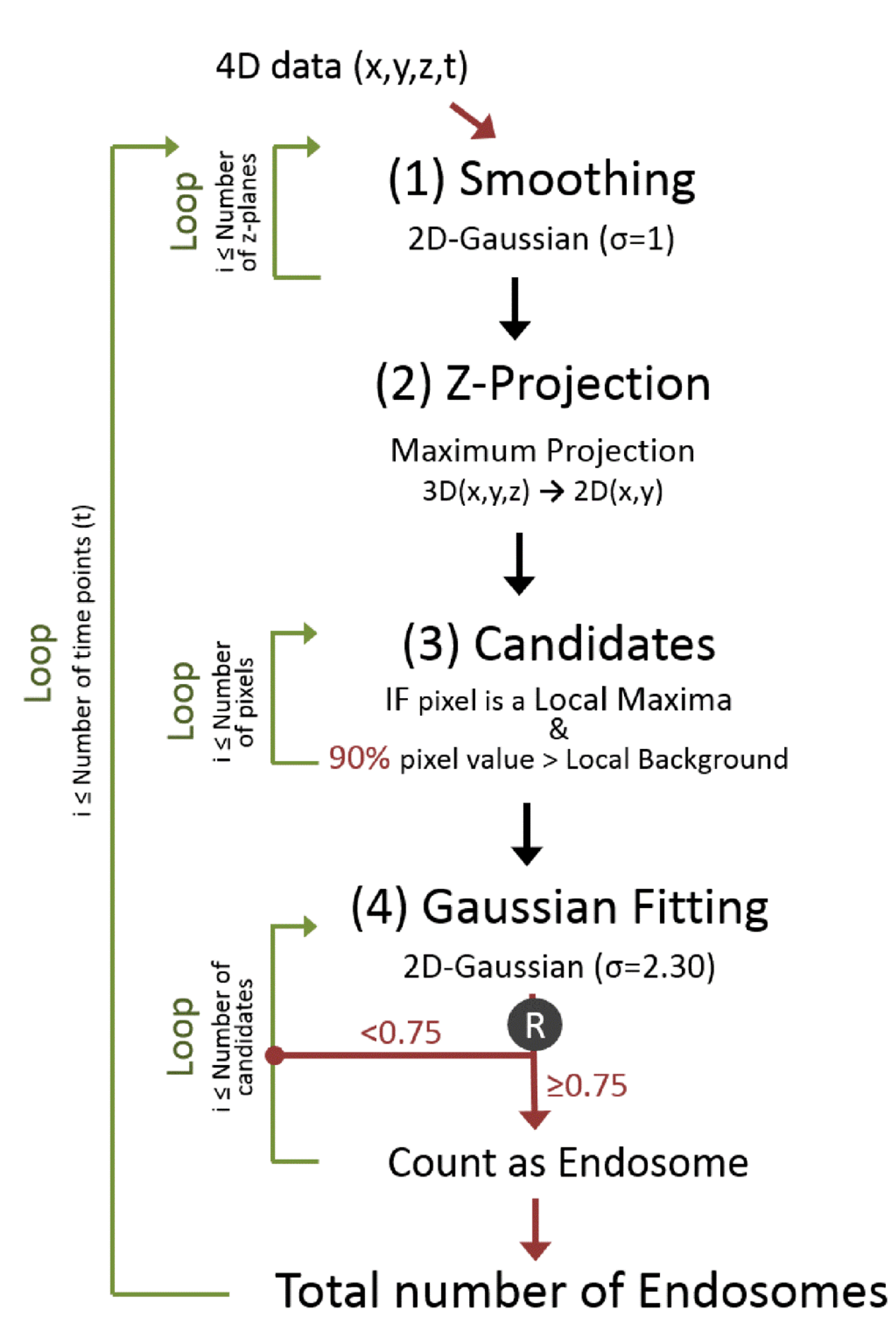

Supplement: S2 Fig — (TIFF) [file pone.0122604.s002.tiff]

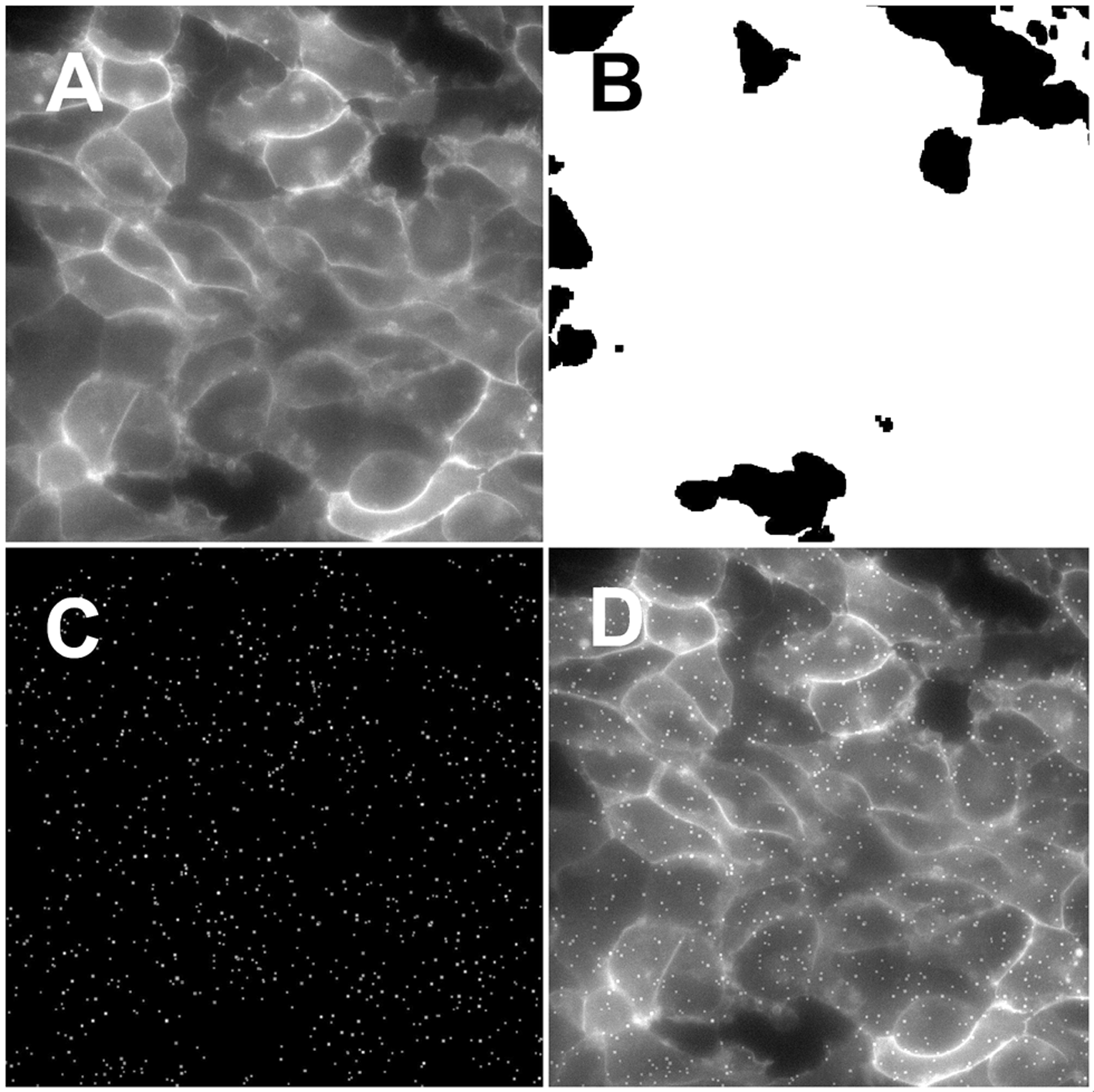

Supplement: S3 Fig — A: Image from untreated cells obtained after Z-stack maximum projection. B: Thresholding of image A to separate those areas containing cells. C: Random distribution of simulated endosomes on these regions corresponding to cells. D: Merging of images A and C. (TIF) [file pone.0122604.s003.tif]

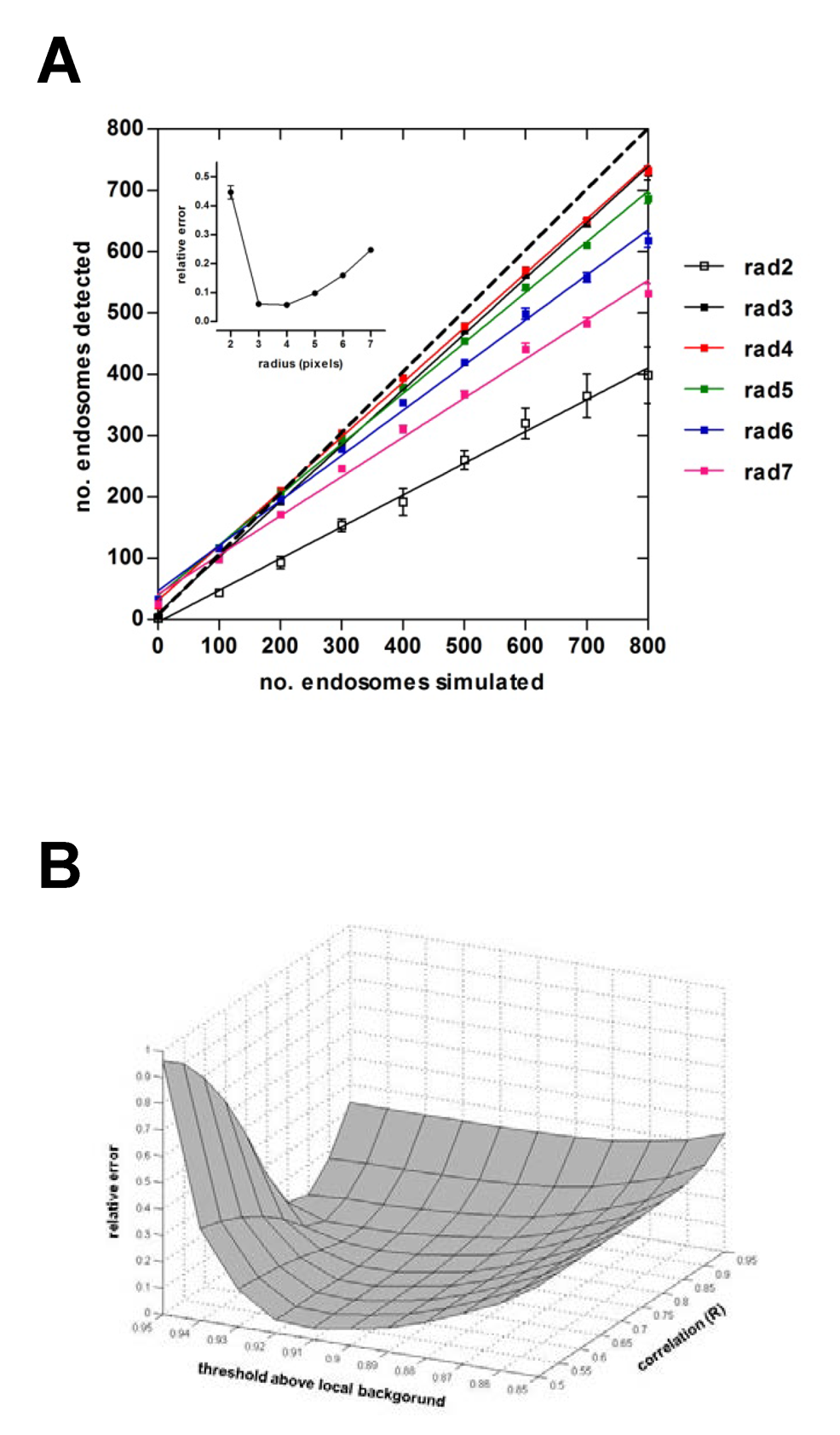

Supplement: S4 Fig — The figure shows the ratio found between the number of detected and simulated endosomes, averaged across 10 independent simulations. Inset graph indicates the relative error between simulated and detected values. B: surface graph showing the relative error found between simulated and detected values from simulations performed by simultaneously modifying values corresponding to threshold above local background and correlation coefficient of the 2D-Gaussian function. (TIFF) [file pone.0122604.s004.tiff]
