# Supplementary material for: Endocytosis as a Biological Response in Receptor Pharmacology: Evaluation by Fluorescence Microscopy
Source: PLoS One. 2015 Apr 7;10(4):e0122604. doi: 10.1371/journal.pone.0122604 (PMC4388511; doi:10.1371/journal.pone.0122604)
Supplement: S1 Q_Endosomes — (HTML) [file pone.0122604.s005.html]

Q\_Endosomes 

## Contents

- Identify tif files in the images\_path folder
- Default optional values
- Read images per time-point and detect endosomes in the following steps:
- Save output files in folder 'Q-endosomes'

```
function endosomes = Q_Endosomes(images_path, options)
```

```
% Q_ENDOSOMES detects and quantifies endosomes
% endosomes = Q_Endosomes(images_path, options)
%
% Example of usage:
%
%   options    = [ ];
%   options.Ntime = 16 ;
%   options.number_cells = 50 ;
%   endosomes  = Q_endosomes('C:\Users\endosomes\2013.03.27 Sample6', options)
%
% INPUT variables:
%
% Required:
%
%     - images_path: computer folder where the images are stored (the images
%       should be placed in a folder with the same name as the images)
%       e.g. images_path = 'C:\Users\endosomes\2013.03.27 Sample6'; in this
%       case, the images would be named: '2013.03.27 Sample6_t01_z01.tif',
%       for consecutive time-points (t) and z-planes (z) (this is the
%       default naming for images exported with ImageJ, using 2 digits)
%
% Optional:
%
%     - options:  structure with parameters that might be changed by the user
%           options.Ntime: number of time-points at which images have been
%               acquired (default: 16)
%           options.pixel_size: pixel size (width, default: 0.13 microm)
%           options.threshold: relative threshold for selecting local maxima
%               candidates (local maxima intensity*threshold > median of
%               neighbouring pixels) (step 3)(default: 0.9)
%           options.radio: radio (in pixels) to select a region around the
%               local maxima pixel (default: 3)
%           options.sigma: sigma (in pixels) of the 2D-Gaussian to be fitted
%               (step 4)(default: 2.3)
%           options.correlation: correlation cut-off value for selecting
%               endosomes in the last step (default: 0.75)
%           options.number_cells: number of cells in the image; if provided,
%               the number of endosomes per cell will be computed, along with
%               the absolute number of endosomes
%           options.figures: 'yes' or 'no', depending on whether the user
%               wants the figures with the results to be displayed and saved
%               (default: 'yes')
%
% OUTPUTS:
%
% All outputs generated by this function are stored in a folder called
% 'Q_endosomes', created inside the images_path folder
%
%     - endosomes: matlab structure (saved as 'results_endosomes.mat'),
%       containing the following fields:
%           endosomes.number: vector (1 x Ntime) with the absolute number of
%               endosomes at each time-point
%           endosomes.number_per_cell: if the number of cells is
%               provided, the number of endosomes per cell in each
%               time-point is also computed
%           endosomes.location: coordinates of each detected endosome
%           endosomes.intensity: mean intensity of endosomes per time-point
%
%     - excel files ('images_name_endosomes_number.xls' and, if the number
%       of cells is provided, 'images_name_endosomes_per_cell.xls')
%       containing the absolute number and the number of endosomes/cell
%       in excel format
%
%     - tif images with the results of the detection for each time-point;
%       the figures show the candidates for being endosomes inside blue
%       circles, and those finally identified as endosomes marked in red
%
% NOTE_1: If you encounter the following error
% ??? Undefined function or method 'Q_endosomes' for input arguments of type 'char'
% you should, first of all, add the path where this function is stored in matlab, e.g.
% >> addpath('C:\Users\endosomes')
%
% NOTE_2: To automatically close all the figures in matlab, you might use
% the following command:
% >> close all
```

## Identify tif files in the images\_path folder

```
cd(images_path);
try temp = strfind(images_path,'/'); end
try temp = strfind(images_path,'\'); end
if length(images_path) == temp(end)
    image_name = images_path(temp(end-1)+1:end-1);
else
    image_name = images_path(temp(end)+1:end);
end
a=ls('*.tif*');
warning('off')
```

```
Error using Q_Endosomes (line 81)
Not enough input arguments.
```

## Default optional values

```
if nargin < 2,                           options = [ ];                       end
if ~isfield(options, 'Ntime'),           options.Ntime = 16;                  end
if ~isfield(options, 'pixel_size'),      options.pixel_size = 0.13;           end
if ~isfield(options, 'threshold'),       options.threshold = 0.9;             end
if ~isfield(options, 'radio'),           options.radio = 3;                   end
if ~isfield(options, 'sigma'),           options.sigma = 2.3;                 end
if ~isfield(options, 'correlation'),     options.correlation = 0.75;          end
if ~isfield(options, 'number_cells'),    options.number_cells = NaN;          end
if ~isfield(options, 'figures'),         options.figures = 'yes';             end


Nt   = options.Ntime;
Nz   = size(a,1)./Nt;
thr = options.threshold;
sigmag = options.sigma;
cor  = options.correlation;
rad = options.radio;
```

## Read images per time-point and detect endosomes in the following steps:

(1) Smoothing the image with a 2D-gaussian (sigma = 1 pixel) (2) Z-projection, taking the maximum value per z-plane (3) Detection of local maxima candidates: if the intensity of a given pixel is higher than its neighbors in a radio of 3 pixels (7x7 matrix), and its intensity\*threshold (default 0.9) is higher than the median value of its neighbors (4) Gaussian fitting: identification of endosomes among the candidates by correlating the above 7x7 region with a 2D-gaussian function of sigma = 2.3 pixels (corresponding to the sigma of an average endosome); the correlation (R) value obtained must be higher than the cut-off R (0.75, by default) (defined in options.correlation)

```
endosomes = [ ];
for t = 1 : Nt
    disp (['----- Processing images acquired at time ' num2str(t) '/' num2str(Nt) ' -----'])
    for z = 1 : Nz
        if t==1 && z==1
            try
                temp = imread([image_name '_t0' num2str(t) '_z0' num2str(z) '.tif']);
                xdim = size(temp,1);
                ydim = size(temp,2);
                d    = zeros(xdim,ydim,Nz);
            catch
                error('Please, images should be named as follows: imagename_t01_z01.tif');
            end
        end

        if z<10 && t<10
            d (:,:,z) = imread([image_name '_t0' num2str(t) '_z0' num2str(z) '.tif']);
        elseif z<10 && t>=10
            d (:,:,z) = imread([image_name '_t' num2str(t) '_z0' num2str(z) '.tif']);
        elseif z>=10 && t<10
            d (:,:,z) = imread([image_name '_t0' num2str(t) '_z' num2str(z) '.tif']);
        elseif z>=10 && t>=10
            d (:,:,z) = imread([image_name '_t' num2str(t) '_z' num2str(z) '.tif']);
        end
    end

    if  round(options.pixel_size*100)./100 ~= 0.13               % Rescale if resolution is different than expected (0.13 microm, pixel width)
        res = round(options.pixel_size*1000);
        res_default = round(0.13*1000);

        d2 = [ ];
        for z = 1 : Nz
            temp = resample(squeeze(d(:,:,z)), res, res_default);
            d2 (:,:,z) = resample(squeeze(temp)', res, res_default)';
        end
        d = d2;
        xdim = size(d,1);
        ydim = size(d,2);
    end

    % (1) Smoothing the image with a 2D-gaussian (sigma = 1 pixel = 0.13 microm)

    dfilt = zeros(size(d));
    gauss_filt = fspecial('gaussian',11,1);
    for z = 1 : Nz
        dfilt(:,:,z) = imfilter(d(:,:,z),gauss_filt,'replicate');
    end

    % (2) Z-Projection, taking the maximum value per z-plane

    dfiltz = max(dfilt,[],3);

    % (3) Identificacion of local maxima candidates

    dmax = zeros(size(dfiltz));
    for i = rad+1 : xdim-(rad+1)
        for j = rad+1 : ydim-(rad+1)
            dneigh = dfiltz(i-rad:i+rad, j-rad:j+rad);    % Neighboring pixels
            dneigh(rad+1,rad+1) = NaN;    % Value of the local maxima set to NaN to avoid influence in the following computation of the median

            if (sum(sum(dfiltz(i,j)>=dneigh)) == (rad*2+1)*(rad*2+1)-1)...    % Intensity of a given pixel must be higher than its neighbors in a radio of 4 pixels
                    && dfiltz(i,j)*thr > nanmedian(dneigh(:))                 % and its intensity*threshold must be higher than the median value of its neighbors
                dmax(i,j)=1;
            end
        end
    end

    [y1,x1] = find(dmax==1);     % Coordinates of local maxima candidates for  endosomes
    disp(['         Number of candidates: ' num2str(length(x1))])

    % (4) Final identification of endosomes, by computing the correlation of the area around the local maxima with the a 2D-gaussian function (sigma = 2.17 pixels)

    gauss_cor = fspecial('gaussian',rad*2+1,sigmag);
    dcor = zeros(size(dfiltz));

    for i = 1 : length(x1)
        rr = [ ];
        try                                                            % Try: if a candidate is at the border of the image, a region around it can not be selected
            dneigh = dfiltz(y1(i)-rad:y1(i)+rad, x1(i)-rad:x1(i)+rad);
            r = corrcoef(dneigh,gauss_cor);
            if r(1,2) >= cor
                dcor(y1(i),x1(i)) = r(1,2);
            end
        end
    end

    [y2,x2] = find(dcor>=cor);    % Coordinates of detected endosomes (candidates with a correlation with a 2D-gaussian > options.correlation)
    disp(['         Number of ENDOSOMES found: ' num2str(length(x2))])

    % Results

    % Output matlab structure: endosomes

    Nendos = length(x2);
    endosomes.number(t) = Nendos;     % Absolute number of endosomes
    if ~isnan(options.number_cells)
        endosomes.number_per_cell(t) = Nendos / options.number_cells;    % Number of endosomes per cell (if number of cells is provided by the user)
    end
    endosomes.location{t} = [x2 y2];    % Endosomes coordinates

    intens = 0;
    for i = 1 : length(x2)
        intens = intens + dfiltz(y2(i),x2(i));
    end
    endosomes.intensity(t) = intens/Nendos;    % Mean intensity of the endosomes per time-point


    % If requested, make and save figures

    if strmatch(options.figures,'yes')
        figure('Color',[1 1 1])
        if t==1
            cmin=min(dfiltz(:))*1.3;
            cmax=max(dfiltz(:))*0.7;
        end
        imshow(dfiltz), caxis([cmin cmax])
        hold on
        blue = plot(x1,y1,'o','MarkerSize',6,'Color',[0 0 0.7]);   % Initial candidates (local maxima): blue circle
        red  = plot(x2,y2,'o','MarkerSize',2,'Color',[0.7 0 0]);   % Finally detected endosomes: red small circle

        if ~isnan(options.number_cells)
            title([image_name '    Time: ' num2str(t) '    Endosomes: ' num2str(Nendos) '  (' num2str(round(Nendos/options.number_cells*10)/10) '/cell)'])
        else
            title([image_name '    Time: ' num2str(t) '    Endosomes: ' num2str(Nendos)])
        end

        try
            cd('Q-Endosomes')
        catch
            mkdir('Q-Endosomes')
            cd('Q-Endosomes')
        end

        print('-dtiff','-r100',[image_name '-Time ' num2str(t) '.tif']);     % Save figures with the results (image resolution might be changed, e.g. '-r300')
        legend([blue,red],'Candidates','ENDOSOMES','Location','NorthEast')   % Legend to be displayed only in matlab figures
        cd(images_path)
    end
end
```

## Save output files in folder 'Q-endosomes'

```
try
    cd('Q-Endosomes')
catch
    mkdir('Q-Endosomes')
    cd('Q-Endosomes')
end

save results_endosomes endosomes      % Results in .mat format
xlswrite([image_name '_endosomes_abs_number.xls'],endosomes.number')     % Results in .xls format (abolute number of endosomes)
if ~isnan(options.number_cells)
    xlswrite([image_name '_endosomes_per_cell.xls'],endosomes.number_per_cell')  % Results in .xls format (number of endosomes/cell)
end

cd(images_path)

disp('---------------------------------------------------------------------------------------------------------------------------------------------')
disp(['Please, check the results in ' pwd '\Q-Endosomes'])
disp('---------------------------------------------------------------------------------------------------------------------------------------------')
```

Published with MATLAB® 7.13
