# Supplementary material for: Endocytosis as a Biological Response in Receptor Pharmacology: Evaluation by Fluorescence Microscopy
Source: PLoS One. 2015 Apr 7;10(4):e0122604. doi: 10.1371/journal.pone.0122604 (PMC4388511; doi:10.1371/journal.pone.0122604)
Supplement: S1 Supplemental Data — (DOCX) [file pone.0122604.s006.docx]

# Supplemental Data

## Endosome Characterization

A previous step in the development of the Q-Endosomes (quantification of endosomes) algorithm was to characterize the critical parameter sigma of the endosomes to be detected. To this end, 195 endosomes were selected by a trained experimenter in images (Z-maximum projection) obtained from cells treated with different opioid agonists for 10 minutes. The coordinates and fluorescence values of a region of 21x21 pixels centered in the local maxima of each endosome were extracted and adjusted to the best possible 2D-Gaussian function either before or after smoothing the image with a Gaussian filter of sigma = 1 pixel (0.13 µm). The characterization of non-smoothed endosomes aimed to calculate the optimal sigma values to be employed in the model to simulate them. These endosomes showed a sigma value of 2.07 ± 0.53 pixels (mean ± SD) and an intensity value of 1652 ± 178 a.f.u. (arbitrary fluorescence units). Furthermore, the characterization of endosomes in smoothed images was devised to determine the optimal 2D-Gaussian function to be fitted in the last step of the algorithm. In this case, the sigma value was 2.30 ± 0.05 pixels (0.30 ± 0.15 µm) and the intensity was 1650 ± 350 (a.f.u.) (S1 Figure A, B).

In order to test whether the sigma value of real endosomes differed from the characteristic sigma of other local maxima in the image –usually located in cell membranes– the above procedure was repeated for 246 regions of 21x21 pixels centered in local maxima of untreated cells (i.e. without endosomes). These regions showed an average sigma value of 5.15 ± 4.96 pixels (0.67 ± 0.23 µm), which was significantly different than the typical endosome sigma value (t-student_439_ = 10.87, p<0.0001) (S1 Figure C).

## Q-Endosomes Algorithm: Method to Detect and Quantify Endosomes

We used Matlab to develop the Q-Endosomes algorithm (S1 Q_Endosomes), aimed at quantifying the number of endosomes formed during treatment with receptor agonists, as illustrated in S2 Figure. The input to the algorithm is four-dimensional data (x,y,z,t), i.e. images in 3D-space acquired at different time points. The method comprises the following four steps, which are recurrently repeated for each time point.

1. *Smoothing*. In order to reduce noise, images at each z-plane are smoothed using a Gaussian kernel of sigma 1 pixel.
2. *Z-Projection*. To reduce the dimensionality of the data, the 3D z-stacks are projected onto a 2D representation by selecting the maximum value across the z-axis.
3. *Local Maxima Candidates*. A pixel is considered a candidate for endosome if it fulfills the following two requirements: first, if it is a local maxima, i.e. its intensity is higher than all the pixels surrounding it in a 3 pixels radio and, second, if its intensity value is 90% above the local background. The local background is defined as the median value of the surrounding pixels. This step ensures that only those local maxima showing a relatively high intensity with respect to the local background are taking into consideration for the next step.
4. *Gaussian Fitting*. The above regions of 7x7 pixels (3 pixel radius; empirically chosen as explained later) around the local maxima candidates are fitted to a 2D-Gaussian function of sigma=2.30 pixels (i.e. the previously estimated sigma value of an average endosome, see above). A candidate local maximum is counted as an endosome if the correlation between the selected region of the image and the 2D-Gaussian function is above 0.75. Otherwise, the local maximum is considered as a false positive. As shown in S1 Figure D, this correlation cut-off value is optimal to differentiate real endosomes from false positives.

## Model to Simulate Endosomes: Assessment of Accuracy of Q-Endosomes

In addition to the algorithm, we developed a model in Matlab to simulate images containing endosomes based on the fluorescence intensity and distribution measured in the sample of real ones. This model allowed us to estimate the accuracy of the Q-Endosomes algorithm.

In order to recreate images containing endosomes, we first took a data set of untreated cells (i.e. with no endosomes) and projected the maximum value across the z-plane, hence obtaining a background image of 1004x1002 pixels (see S3 Figure A). In this image we selected only those pixels with intensity values above a given threshold, corresponding to areas containing cells (S3 Figure B). Next, endosomes were modelled as 2D-Gaussian objects with the same features of the previously measured non-smoothed endosomes (sigma=2.07 ± 0.53 pixels, and intensity=1652 ± 178 a.f.u.). A known number of simulated endosomes, ranging from 0 to 800, were randomly distributed over the regions in the image containing cells, as determined in the previous thresholding step (S3 Figure C). Finally, both the background image and the image comprising the simulated endosomes were merged to create the model (S3 Figure D). Thus, the model provided a known number of endosomes while recreating critical features of real data (e.g. cell membranes with high fluorescence intensity present in the image and endosomes located inside cells).

This model was used to empirically determine the size of the region to be fitted, as this might have an influence on the results. We performed 10 virtual experiments per radius to be tested (from 2 to 7 pixels, corresponding to 0.26 to 0.91 µm). Our results indicate that the highest accuracy was obtained by using a radius from 3 to 4 pixels (i.e. a 7x7 pixels region) (S4 Figure A). The minimum relative error found between the number of endosomes detected and the number of endosomes simulated corresponded to radius values of 3 and 4 (S4 Figure A inset).

Once the radius value was fixed, additional simulations were carried out by modifying simultaneously the values corresponding to the threshold above the local background and the correlation coefficient (R) of the 2D-Gaussian function (S4 Figure B). According to the minimum relative error found, 90% (0.9) and 0.75 were adopted for these variables respectively in subsequent analysis.

Overall, the accuracy of the algorithm was assessed by using this simulation model. The proportion of correctly identified endosomes was higher than 90% when the number of simulated endosomes ranged from 0 to 800. (S4 Figure).
